# Objective assessment of gait and posture symptoms in Parkinson’s disease using wearable sensors and machine learning

**DOI:** 10.3389/fnagi.2025.1618764

**Published:** 2025-08-08

**Authors:** Lingyan Ma, Shinuan Lin, Jianing Jin, Zhan Wang, Xuemei Wang, Zhonglue Chen, Yun Ling, Fei Zhang, Kang Ren, Tao Feng

**Affiliations:** ^1^Center for Movement Disorders, Department of Neurology, Beijing Tiantan Hospital, Capital Medical University, Beijing, China; ^2^China National Clinical Research Center for Neurological Diseases, Beijing Tiantan Hospital, Capital Medical University, Beijing, China; ^3^GYENNO Science Co., Ltd., Shenzhen, China; ^4^HUST—GYENNO CNS Intelligent Digital Medicine Technology Center, School of Artificial Intelligence and Automation, Huazhong University of Science and Technology, Wuhan, China; ^5^Parkinson's Disease Center, Beijing Institute for Brain Disorders, Beijing, China

**Keywords:** Parkinson’s disease, gait, posture, walking assessment, wearable sensor

## Abstract

**Objective:**

Gait and posture symptoms—such as gait impairments, postural instability, and posture deformations—are common in Parkinson’s disease (PD) and closely linked to falls. Traditional assessments using clinical scales are time-consuming and prone to subjective bias. This study aims to predict the severity of gait and posture symptoms using data collected from wearable sensors during a single laboratory-based walking assessment, providing an objective, efficient, and automated evaluation approach.

**Methods:**

Sensor-based gait parameters were collected from 225 PD participants (mean age 63.15 ± 10.46 years) through a standardized walking assessment. The dataset was randomly split into a training set (80%) and an independent test set (20%) with balanced age, sex, and PD duration. Two machine learning models—extreme gradient boosting (XGBoost) and support vector machine (SVM)—were trained to predict scores for five gait and posture items (#3.9–3.13) from the MDS-UPDRS III.

**Results:**

XGBoost was chosen as the final model due to its better performance than SVM. Across all five gait and posture items, the models achieved over 80% acceptable accuracy. For items #3.9–#3.11, absolute accuracy surpassed 70%, and macro-F1 scores were above 0.60 in leave-one-out cross-validation (LOOCV). The model’s performance on the independent test set matched LOOCV results, confirming robustness. A total of 35, 35, 30, 30, and 40 gait features were selected for the predictive models of items #3.9–#3.13, respectively. Among these, key features with significant clinical relevance were identified. For example, *Effective Trial Duration* (R = 0.522, *p* < 0.001) had a positive correlation, while *Shank—Swing RoM—mean (max)* (R = −0.629, *p* < 0.001) had a negative correlation with scores on item #3.10. In addition, *180° Turn—Steps – mean* (R = 0.482, *p* < 0.001) had a positive correlation with scores on item #3.11. These findings align with known clinical manifestations, reinforcing the clinical relevance of the identified gait features.

**Conclusion:**

This study demonstrates the feasibility of using wearable sensor data to objectively assess gait and posture symptoms in PD. Though conducted in a clinical setting, the approach may support clinicians through consistent assessments and more frequent monitoring, with potential for future home-based use to enable longitudinal symptom tracking.

## 1 Introduction

Parkinson’s disease (PD) is the second most common neurodegenerative disorder, with its prevalence rising significantly over the past three decades (Kalia and Lang, 2015; Su et al., 2025). Gait and posture symptoms—such as gait impairments, postural instability, and posture deformities—are common symptoms in PD and serve as crucial indicators of disease progression and fall risk (Lau et al., 2019; Debû et al., 2018). Gait and posture disturbance are also associated with non-motor issues, including anxiety and cognitive decline (Thenganatt and Jankovic, 2014; Artigas et al., 2022). As PD is a progressive disease (Van der Marck et al., 2009), gait and posture symptoms worsen over time, resulting in a significant decline in patients’ mobility and independence, thereby affecting their overall quality of life (O'Gorman Tuura et al., 2018).

Currently, gait and posture symptoms are primarily assessed using the five items from the Movement Disorder Society’s Unified Parkinson’s Disease Rating Scale (MDS-UPDRS III) (Goetz et al., 2008) —#3.9 (arising from a chair), #3.10 (gait), #3.11 (freezing of gait), #3.12 (postural stability), and #3.13 (posture). These items collectively capture key aspects of axial motor function, including standing up, walking performance, freezing episodes, postural stability, and overall posture. Together, these tasks reflect critical dimensions of gait and posture control, which are particularly relevant for evaluating gait disturbances, postural instability, and overall functional mobility in individuals with PD. The evaluation of these items requires multiple motor tasks and relies heavily on subjective clinician judgment, which presents several limitations: (1) it imposes a significant burden on both patients and clinicians, (2) it is inherently subjective, leading to inter-rater variability (Zogaan et al., 2024; Stebbins et al., 2013) and (3) subtle motor symptoms, such as speech, low-amplitude tremor, and axial symptoms, may be difficult to detect through visual observation alone (Zogaan et al., 2024; Stebbins et al., 2013; De Rose et al., 2012). These limitations highlight the need for complementary assessment approaches that are objective, efficient, and capable of capturing subtle motor abnormalities.

Recent advances in wearable sensor technology, particularly the development of wireless inertial measurement units (IMUs) with high sampling frequency and improved measurement accuracy, have enabled objective, quantitative assessment of gait impairments in PD (Moreau et al., 2023). These technological improvements allow for continuous, high-resolution, and non-invasive monitoring of gait and posture, addressing limitations of traditional clinical tools such as the MDS-UPDRS, which often fail to detect subtle motor abnormalities, particularly those related to gait and posture. In parallel, machine learning models have shown promise in analyzing complex sensor-derived kinematic data, facilitating the detection of clinically relevant motor fluctuations. Several studies have demonstrated the feasibility of using sensor-based measurements to predict scores on gait and posture clinical scales. For example, Abujrida et al. (2020) predicted scores for MDS-UPDRS II items #2.12 (walking and balance) and #2.13 (freezing) using gait parameters collected from a smartphone placed in the front pocket of participants during walking tasks, while Safarpour et al. (2022) utilized gait parameters obtained from wearable sensors placed on each foot and the lower lumbar region of participants during two standing balance tasks in a laboratory setting and daily activities at home to estimate postural instability gait difficulty (PIGD) scores. While these studies demonstrate the feasibility of sensor-based PD assessment, they present notable limitations: small sample sizes (e.g., fewer than 40 participants), reliance on self-recorded data with limited standardization, and a narrow focus on a single or aggregated symptom score (e.g., PIGD), which limits their ability to provide item-level assessment of specific gait and posture symptoms.

To address these gaps, this study aims to develop a robust, machine learning-based predictive model using wearable sensor-derived kinematic features to estimate scores for all five individual gait and posture items of the MDS-UPDRS III (#3.9–#3.13) from a single, standardized walking assessment. By providing item-level, objective, and efficient symptom evaluation, this approach offers a more comprehensive alternative to conventional, subjective clinical assessments of gait and posture in PD.

## 2 Materials and methods

### 2.1 Participants

This study was approved by the Ethics Committee of Beijing Tiantan Hospital. Written informed consent was obtained from all the participants. A total of 248 participants diagnosed with PD (mean age: 63.46 ± 10.54 years) were recruited from Beijing Tiantan Hospital, Capital Medical University. Participants met the diagnostic criteria for PD established by the Movement Disorder Society (MDS) (Postuma et al., 2015). The exclusion criteria were as follows: (1) a history of stroke and cerebrovascular disease, (2) orthopedic impairment or other disease which may lead to gait disturbance, (3) MDS-UPDRS III 3.10: gait is score 4, (4) cognitive disorder was evaluated using Mini-mental State Examination (MMSE) and the cutoff values of MMSE for exclusion were adjusted by the education level where <18 for illiterate level, <21 for elementary level, and <24 for middle or above level (Katzman et al., 1988). Of these, video recordings of MDS-UPDRS III gait and posture-related items (#3.9–#3.13) were available for 225 participants and used for additional multi-rater scoring.

### 2.2 Setting and design

**Figure 1:**
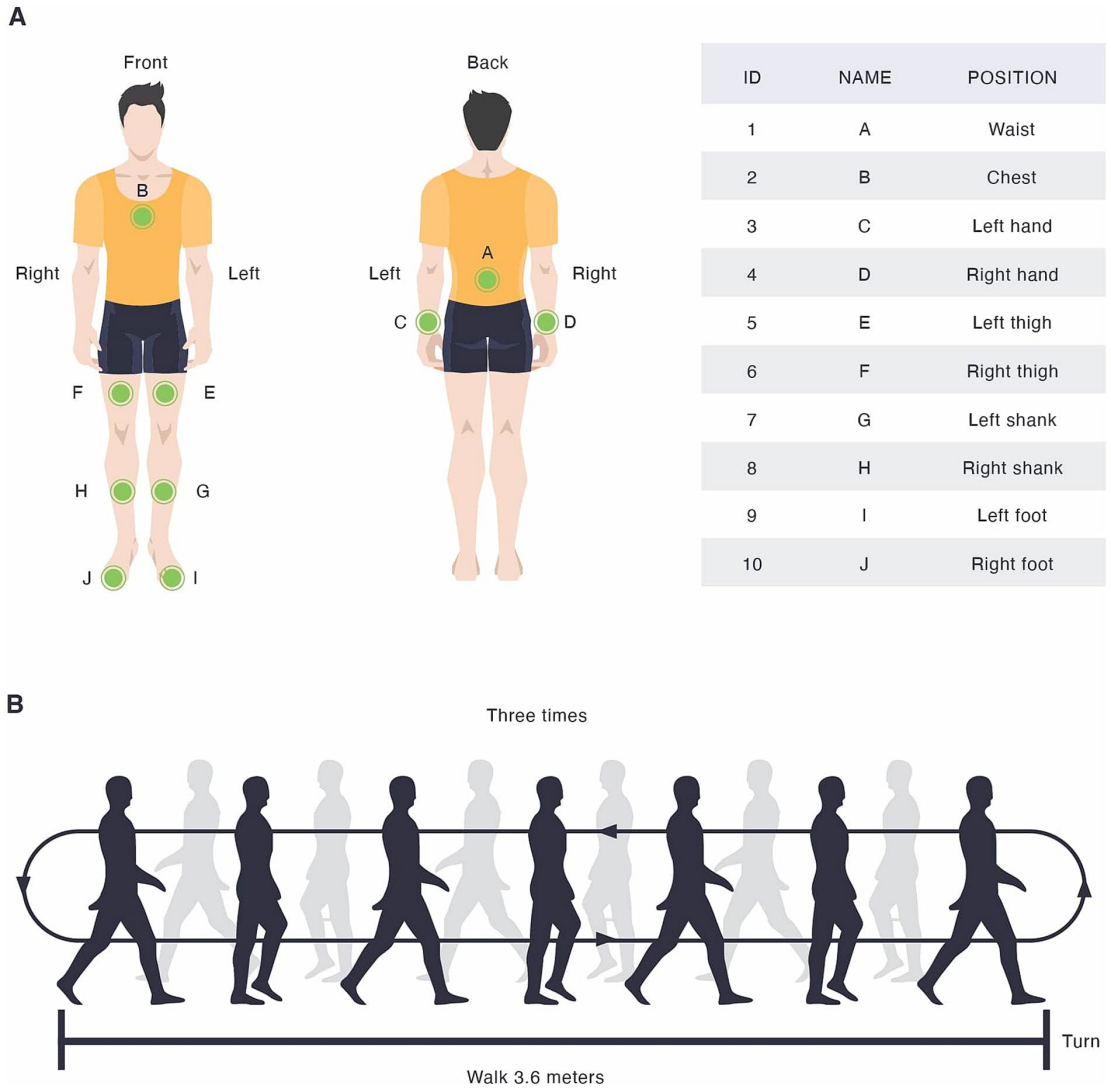
**(A)** Sensor locations and **(B)** walking assessment.

The MMSE and MDS-UPDRS III were administered and scored by a movement disorder specialist. An Motor Function and Motor Symptom Quantitative Assessment System (GYENNO SCIENCE, Shenzhen, China) (GYENNO Technologies Co. Ltd., 2022) was used in this assessment. This wearable motion and gait quantification assessment system is approved by Conformitè Europëenne Medical (CE Medical), National Medical Products administration (NMPA), and U.S. Food and Drug Administration (FDA). Moreover, this assessment platform has also supported research efforts at the intersection of medicine and engineering (Cai et al., 2023; He et al., 2024; Zhang et al., 2024; Lin et al., 2023). Participants performed a standardized gait assessment consisting of three consecutive trials, referred to as shuttle walk tests. Each trial required participants to walk straight along a 3.6-meter path, to execute a 180-degree turn, and return to the start position, while wearing ten inertial measurement unit (IMU) sensors (Figure 1). Two sensors were secured to the dorsal side of each wrist. The chest sensor was positioned over the sternum, while the waist sensor was placed at the level of the fifth lumbar vertebra (L5). For the lower limbs, a pair of thigh sensors were attached bilaterally, 7 cm above the knee, and a pair of shank sensors were positioned 7 cm below the knee joints. In addition, two foot sensors were fixed on the dorsal side of the metatarsus (instep) of each foot. All sensors were fastened firmly at their respective positions using adjustable straps. The 3.6-meter distance was selected due to its widespread use in Parkinson’s disease gait assessments, offering an optimal balance between patient safety, spatial feasibility in clinical environments, and its demonstrated ability to effectively provoke early gait abnormalities and freezing episodes (Stebbins et al., 2013; Choi et al., 2020).

For 225 participants with available video recordings of gait and posture-related items (#3.9–#3.13) during the MDS-UPDRS III assessment, a multi-rater, multi-round adjudication process was employed to minimize subjectivity and inter-rater variability. Two qualified raters independently assessed each video. For items where ratings differed, a consensus meeting was held after 1 week to re-evaluate and discuss. If consensus matched either original rating, it was adopted as the final score. If not, a third, more senior movement disorder specialist conducted an independent assessment, which was used as the final rating. Our analysis was conducted based on participants for whom video recordings were available, with the finalized re-rated scores from the adjudication process serving as the definitive ground truth labels.

### 2.3 Sensor measurements

Each IMU sensor consisted of 3-axial accelerometers and gyroscopes. Sensor data from the accelerometers and gyroscopes (x, y, and z-axis values) were continuously captured at a frequency of 100 Hz by the ten IMU sensors throughout the gait assessments in real time and were transmitted to the host computer via a Bluetooth link. The gait assessment was segmented into two types of phases: straight walking and turning, following the approach described in our previous studies (Lin et al., 2023). These phases were identified using kinematic signals from the waist, specifically the waist’s horizontal rotation angle. Distinct peaks in the waist rotation curve correspond to turning events. The first and second peaks indicate the onset and completion of the first turn, while the third and fourth peaks mark the start and end of the second turn. The remaining periods were classified as straight walking phases. Before feature extraction, the raw sensor data underwent a preprocessing pipeline. Specifically, the data were processed through a filtering procedure to reduce noise while preserving the true motion signals. Subsequently, orientation estimation was performed to convert the raw acceleration and angular velocity signals into meaningful spatial kinematic parameters (e.g., joint angles and angular velocities).

Based on the processed sensor data, a total of 240 kinematic features, such as *Step Length*, *Cadence*, and *Double Support*, were calculated. The definitions of these kinematic features are listed in Supplementary Table 1. Each participant completed three walking trials. For each trial, the walking assessment was segmented into two straight-walk sections and two turning sections, resulting in a total of six straight-walk sections and six turning sections per participant across all three trials, refer to straight-walk I (SW-1), 180° Turn I (T1), SW-2, T2, SW-3, T3, … and SW-6, T6. Extracted gait features were divided into three categories: (1) Segmentation-gait cycle-based features: these features were calculated within individual gait cycles but only for straight-walk sections. Within each straight-walk section, gait features were calculated for each gait cycle and then averaged across all gait cycles within that section. Averaging across all gait cycles in each section helps to minimize the impact of stride-to-stride variability and random fluctuations, providing a more stable and reliable estimate of each gait parameter for that section. By analyzing each section separately rather than only across the entire assessment, we were able to capture detailed, gait characteristics, which may be clinically relevant but could be masked if only whole-trial averages were considered. Gait cycles were detected by initial contact (IC) and terminal contact (TC) events as described in our previous studies (Lin et al., 2023). The right gait cycle begins from the right IC, then right TC, and then the right IC. The left gait cycle begins from the left IC, then the left TC, and then the left IC. Salarian et al. (2004) demonstrated that shank gyroscope signals are particularly effective for identifying IC and TC events during walking. Specifically, the first local minimum before and after each peak in the shank’s angular velocity was used to determine the timing of IC and TC events. Following a similar approach, we extracted IC and TC events from our data. For example, *Trunk—Max Sagittal Angular Velocity*, which was the measurement of the sagittal projection of the torso’s maximum angular velocity, it was calculated within individual gait cycles, and the values were then averaged across all gait cycles within a straight-walk section. (2) Segmentation features: this type of gait feature was calculated directly within each section, independent of gait cycle detection. These features reflect the overall performance or characteristics of an entire section (e.g., a straight-walk or turning segment) without relying on identifying precise gait events such as IC or TC. By considering the specific conditions of each section, segmentation features allow for a more comprehensive assessment of gait and posture characteristics during different phases of the assessment, providing complementary information beyond gait cycle-based features. For example, *SW—Lumbar—Difference of Sagittal Sway* was the difference of the sagittal projection of the waist’s tilt relative to the gravity vertical between the start and end moment of a straight walk section. (3) Whole assessment features: this type of gait features was calculated across the entire gait assessment, providing a global summary of gait performance. For example, *WT – Trunk – Difference of Coronal Sway* represents the difference in the trunk’s coronal tilt relative to the gravity vertical between the start and end of the whole trial. This approach captures overall changes or trends in gait characteristics throughout the entire assessment, allowing for a comprehensive evaluation of general gait stability and performance.

### 2.4 Feature construction

To represent overall gait characteristics, account for differences across straight-walk sections and across turning sections, and minimize bias due to limb dominance, feature construction was performed in two steps. Step1: For both segmentation-gait cycle-based features and segmentation features, the maximum, minimum, mean, and mean of difference across the six straight-walk sections and six turning sections were calculated. As a result, for each parameter, four summary features were generated, noted as—max, —min, —mean, and —diff_mean, respectively. For example, after feature construction step 1, feature *Gait Speed L* was replaced by *Gait Speed L—max, Gait Speed L—min, Gait Speed L—mean, and Gait Speed L—diff_mean*. Step 2: To quantify asymmetry and overall condition, for each pair of left- and right-side parameters, the maximum, minimum, and absolute difference were calculated, resulting in three representative features: (min), (max), and (diff). For example, after feature construction step 2, the pair of left- and right-side parameters *Gait Speed L—mean* and *Gait Speed R—mean* were replaced by *Gait Speed—mean (max), Gait Speed—mean (min),* and *Gait Speed—mean (diff).* This feature construction process was applied based on the features obtained from all three walking trials, ensuring that the final set of representative features for each participant integrated information from all trials and all relevant sections. This strategy enhances the stability and robustness of the extracted gait features while preserving section-level and side-specific information.

### 2.5 Model construction and evaluation

#### 2.5.1 Training and independent test data split

For model development, the dataset comprised 225 participants for whom multi-rater consensus ratings of the MDS-UPDRS III gait and posture-related items (#3.9–#3.13) were available and used as ground truth labels. The dataset was randomly split into 80% for training and 20% for testing, ensuring age, sex, and PD duration were matched between the sets. As a result of this participant-level split, it is difficult to strictly guarantee a perfectly balanced score distribution for each MDS-UPDRS III item between the training and test sets. However, to ensure that the training set contained all available score levels (e.g., 0–4) for each of the five gait and posture items (#3.9–#3.13), we performed repeated random splitting until all score categories were present in the training set for each item. This approach follows recommended practices in clinical machine learning to avoid missing outcome categories during model development. Feature selection and model construction were conducted using the training data. Leave-one-out cross-validation (LOOCV) was performed as a validation method in the training data to fine-tune the model hyperparameters and estimate the model performance. Independent test data were then used to evaluate the final models that were constructed using training data.

#### 2.5.2 Predictive model for scores on the MDS-UPDRS III gait and posture items

Each of the MDS-UPDRS III gait and posture items was rated on a 5-point scale (0 to 4) by a movement disorder specialist. Each item score was transformed into an M-level categorical variable, where the value of M for a specific gait and posture item was determined by the sample size of each level for that specific gait and posture item. If a score level had a sample size≤ 5 on the training data, it was merged into the previous level to create a new level because sample imbalance among levels would bias the overall model. For example, item #3.11 had ≤ 5 samples scoring 4 points and 3 points, and these samples were combined with those scoring 2 on this item, resulting in a final 3-level categorical variable: 0, 1, and combined 2/3/4, Thus, scores on this item would be converted into a 3-level categorical variable (0, 1, or 2/3/4). Item #3.13 had ≤ 5 samples scoring 4 points, and these samples were combined with those scoring 3 on this item, resulting in a final 4-level categorical variable: 0, 1, 2, and combined 3/4. Thus, scores on this item would be converted into a 4-level categorical variable (0, 1, 2, or 3/4). This merging was regarded as an inherent limitation due to sample imbalance. Same score categories were made in independent test data as training data.

To identify the optimal classifier for this study, we compared two models: extreme gradient boosting (XGBoost) (Chen and Guestrin, 2016) and support vector machine (SVM). XGBoost, as an ensemble learning algorithm, is well-suited for capturing complex non-linear relationships among features and offers robust performance with effective built-in feature selection based on the Gain metric, where higher Gain values indicate greater feature importance (Burnwal and Jaiswal, 2023). SVM identifies an optimal separating hyperplane by maximizing the margin between classes. To handle non-linear relationships, it employs kernel functions—such as the Radial Basis Function (RBF)—to transform data from its original low-dimensional space into a higher-dimensional feature space. This transformation increases the likelihood that complex patterns become linearly separable. With an appropriately chosen kernel, this approach enables effective classification even for intricate data distributions (Noble, 2006). Based on these strengths, we implemented both models to determine which would better capture the associations between gait parameters and clinical scores in our dataset.

For feature selection, features were ranked by their Gain scores from the XGBoost model and incrementally incorporated into model construction. Features were ordered from most to least important according to their respective Gain scores. For predictive model construction, the top K features with the highest Gain values were incrementally incorporated—starting from the top 5 features (K = 5) and increasing in steps of five (i.e., K = 5, 10, 15, …, 50). The use of a step size of five provided a balance between performance resolution and computational efficiency. The upper limit of 50 features was set considering the total sample size (*n* = 225) to reduce the risk of overfitting and to maintain model generalizability.

At each feature configuration (each value of K), hyperparameter tuning was conducted via grid search within the training set during cross-validation. For XGBoost, the parameter grid comprised learning rates of 0.05 and 0.1, maximum tree depths of 3 and 4, gamma values of 0.1 and 0.2, and lambda values of 3, 4, and 5. These ranges were selected to balance model complexity and overfitting risk. For the SVM with a RBF kernel, the hyperparameter grid included gamma values of 0.001, 0.01, 0.1, and 1 and penalty parameters (C) of 0.1, 1, 10, and 100. These values were chosen to ensure a broad search over possible decision boundary smoothness and margin settings, as smaller gamma or C values reduce overfitting risk but may underfit, while larger values allow more complex, potentially overfitted models.

For model training and evaluation, LOOCV was applied. Class imbalance, particularly the underrepresentation of severe UPDRS scores, can lead to biased model performance by causing poor sensitivity to minority classes and overfitting to majority classes. To mitigate this issue, the Synthetic Minority Over-sampling Technique (SMOTE) (Branco et al., 2016) was performed within each LOOCV iteration. Specifically, SMOTE was applied only to the training subset of each fold to synthetically generate new samples from the minority classes, ensuring that the left-out test sample remained completely independent of the oversampling process. This strategy effectively reduces class imbalance while avoiding information leakage.

Across all combinations of feature counts, hyperparameters, and model types, the model yielding the highest LOOCV performance (e.g., accuracy and weighted F1) was selected as the optimal configuration. This optimal model, with its corresponding selected features and tuned hyperparameters, was then retrained on the entire training set using the same SMOTE procedure. The final trained model was subsequently applied to the independent test set for unbiased performance evaluation.

#### 2.5.3 Predictive model for scores on the MDS-UPDRS III gait and posture subscale

The score on the MDS-UPDRS III gait and posture subscale was defined as the sum of scores on the five gait and posture items. Least absolute shrinkage and selection operator (LASSO) (Santosa and Symes, 1986; Tibshirani, 1996) was applied in our study to predict the MDS-UPDRS III gait and posture subscale. The regularization constant, lambda, was obtained through 10-fold cross-validation of LASSO which could give the minimum mean cross-validated error. Features were selected using the LASSO algorithm based on the optimal lambda determined previously. Only the features which have non-zero coefficients, beta, were kept as the features for constructing the predictive model for gait and posture subscale.

#### 2.5.4 Model performance evaluation metrics

The performance of the gait and posture item classification models was comprehensively evaluated using several metrics, including weighted F1 score, absolute accuracy (ACC ± 0), acceptable accuracy (ACC ± 1), Cohen’s weighted kappa (Kw), and per-class precision, recall, and F1 score. In this study, ACC ± 0 refers to the proportion of cases where the predicted score exactly matches the true score, while ACC ± 1 reflects the proportion of cases where the absolute difference between the predicted and true scores is ≤1. Per-class precision measures the model’s accuracy in correctly classifying instances of a given class, while per-class recall measures the model’s ability to detect all actual instances within that class. The F1 score for a given class is the harmonic mean of precision and recall, providing a balanced measure of a model’s performance for that class. For example, precision for a give class “Score *0*” is the fraction of instances correctly classified as Score *0* out of all instances the model predicted to belong to Score *0*. Recall for a give class “Score *0*” is the fraction of instances in Score *0* that the model correctly classified out of all instances in Score *0*. The weighted F1 score is calculated as the sum of the F1 scores for each class, weighted by the number of true instances in each class (known as the support), divided by the total number of instances across all classes. Weighted F1 is suitable for imbalanced datasets as it incorporates per-class F1 scores proportionally to the class distribution, providing a performance metric that reflects both model effectiveness and the true class balance, without overly exaggerating the impact of minority classes. The macro-F1 score is ideal for scenarios where fairness across classes matters more than overall accuracy. It highlights model performance on underrepresented classes, making it a critical metric for imbalanced datasets. K*w* (Cohen, 1968) measures agreement between predicted and actual scores, applying higher weights to greater disagreements. The value of *Kw* was interpreted as follows (Landis and Koch, 1977): <0.00, poor agreement; 0.00–0.20, slight agreement; 0.21–0.40, fair agreement; 0.41–0.60, moderate agreement; 0.61–0.80, substantial agreement; and 0.81–1.00, almost perfect agreement.

The performance of the model for predicting the gait and posture subscale score was evaluated in terms of the mean absolute error (*MAE*), root mean square error (*RMSE*) and Spearman correlation coefficient (*R*). The value of *R* was interpreted as follows (Schober et al., 2018): 0.00–0.10, negligible correlation; 0.10–0.39, weak correlation; 0.40–0.69, moderate correlation; 0.70–0.89, strong correlation; and 0.90–1.00, very strong correlation. The MAE and RMSE were calculated as follows:


$$\mathrm{MAE}=\frac{1}{n}\sum_{i=1}^{n}|y_{i}-{\hat{y}}_{i}|$$

$\text{RMSE}=\sqrt{\frac{1}{n}\sum_{i=1}^{n}{\left({\hat{y}}_{i}-y_{i}\right)}^{2}}$, where $y_{i}$ is the true score and ${\hat{y}}_{i}$ is the predicted score.

Spearman’s correlation was used instead of Pearson’s correlation because the assumption of normality was not met, making a non-parametric approach more appropriate for assessing our data.

#### 2.5.5 Contribution of sensors to each gait and posture item model

The contribution of a specific sensor for an gait and posture item model was defined as the proportion of features derived from that sensor out of the total number of features included in the final gait and posture item model on training data. We grouped the left- and right-side sensors at each location, resulting in sensors at the following location: waist, chest, hand (left/right), thigh (left/right), shank (left/right), and foot (left/right).

## 3 Results

### 3.1 Participants

**Figure 2:**
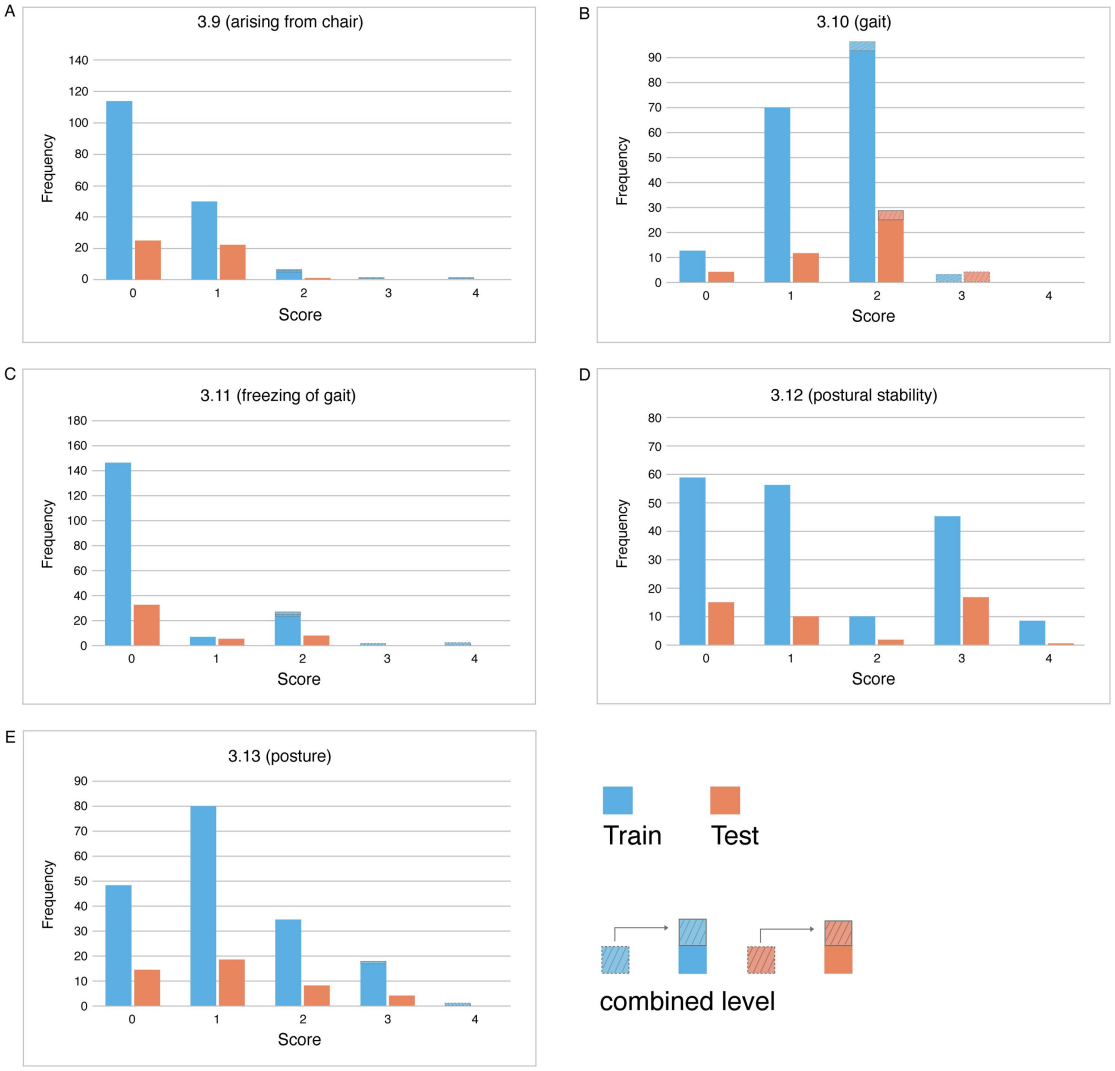
Frequency histogram of the five gait and posture items. Bar charts labeled A to E display frequency distributions for various scores on different activities: **(A)** arising from a chair, **(B)** gait, **(C)** freezing of gait, **(D)** postural stability, and **(E)** posture. Blue bars represent training data, and orange bars represent test data. The legend clarifies the color codes for training and test datasets, as well as a combined level.

**Figure 3:**
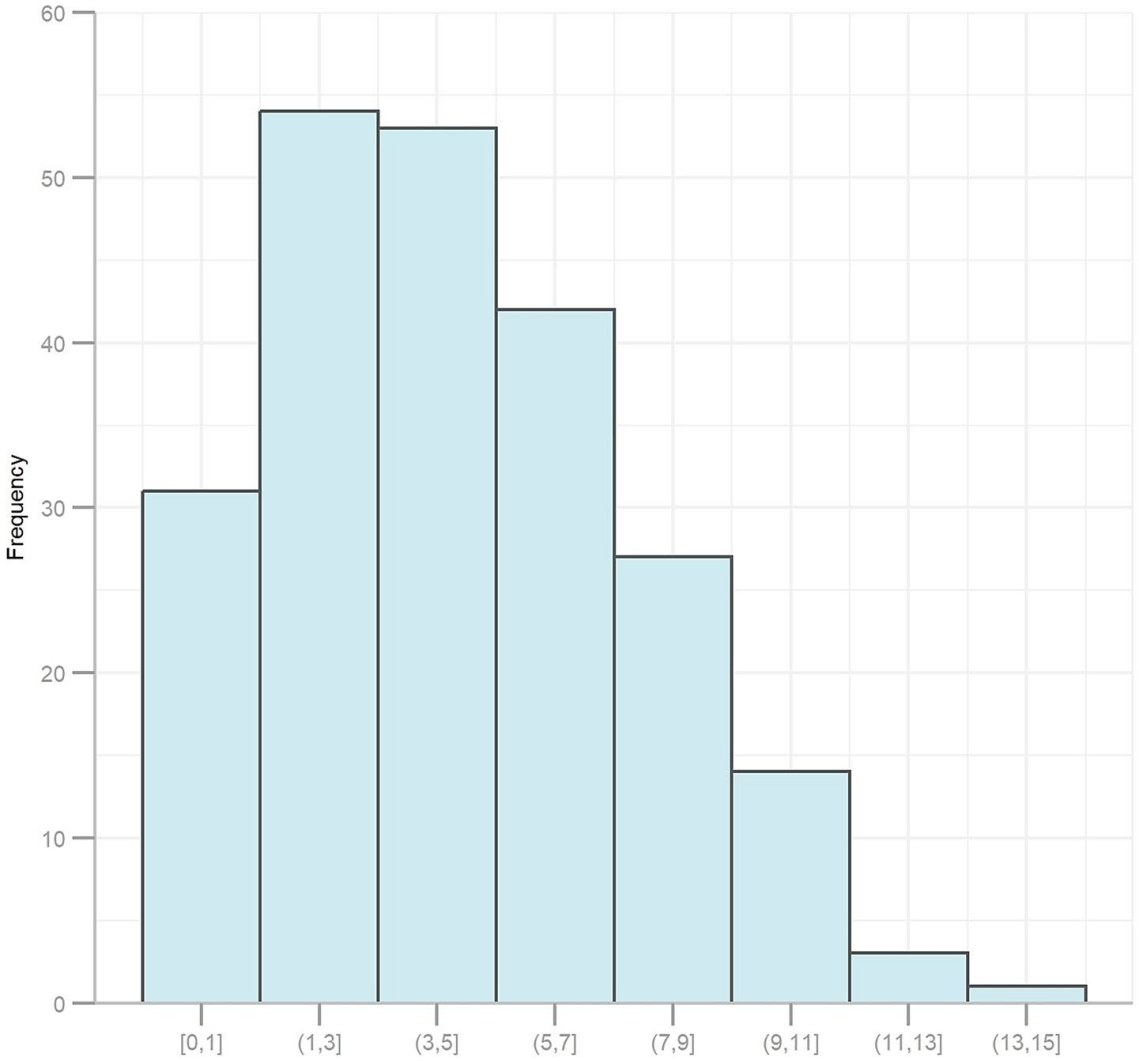
Distribution of participant scores on the MDS-UPDRS III gait and posture subscale.

The primary demographic characteristics of all participants (*n* = 248) and re-rating sample (*n* = 225) are summarized in Table 1. There were no statistically significant differences observed in demographic variables between the overall cohort and the training and testing subsets, for both the original and re-rating samples. Figure 2 presents frequency histogram of scores for each gait and posture item. As described in the Methods section, merged categories (scores *0*, *1*, *2/3/4*) were applied for items #3.9 (arising from chair) and #3.11 (freezing of gait), merged categories (scores *0*, *1*, *2/3*) were applied for items #3.10 (gait), while merged categories (scores *0*, *1*, *2*, *3/4*) were applied for items #3.13 (posture). Figure 3 illustrates the distribution of scores on the MDS-UPDRS III gait and posture subscale, indicating fewer participants with higher scores.

**Table 1:** Demographic characteristics of the participants.

Variables	Original sample				Re-rating sample			
	Overall	Train	Test	p*	Overall	Train	Test	p*
Participants, n	248	198	50		225	180	45	
Age, mean (SD), years	63.46 (10.54)	63.43 (10.72)	63.58 (9.91)	0.996	63.15 (10.46)	62.83 (10.63)	64.39 (9.80)	0.671
Sex: Female, *n* (%)	117 (47.2)	93 (47.0)	24 (48.0)	0.992	106 (47.1)	84 (46.7)	22 (48.9)	0.965
MMSE score, mean (SD)	27.23 (2.37)	27.16 (2.39)	27.54 (2.32)	0.594	27.27 (2.42)	27.22 (2.46)	27.47 (2.30)	0.826
PD duration, mean (SD), years	6.74 (4.22)	6.82 (4.27)	6.39 (4.04)	0.812	6.54 (4.19)	6.56 (4.23)	6.46 (4.04)	0.989
MDS-UPDRS III total score, mean (SD)	32.45 (15.13)	32.31 (15.50)	33.00 (13.69)	0.96	31.74 (14.68)	31.52 (14.27)	32.64 (16.40)	0.904
HY, mean (SD)	2.46 (0.84)	2.45 (0.86)	2.51 (0.74)	0.894	2.39 (0.81)	2.39 (0.77)	2.38 (0.95)	0.997
Medication on: yes, *n* (%)	97 (39.4)	79 (39.9)	18 (37.5)	0.955	90 (40.4)	73 (41.0)	17 (37.8)	0.925
Education^a^: no. (%)				0.532				0.992
Elementary level	35 (14.1)	29 (14.6)	6 (12.0)		33 (14.7)	26 (14.4)	7 (15.6)	
Illiterate level	11 (4.4)	10 (5.1)	1 (2.0)		11 (4.9)	9 (5.0)	2 (4.4)	
Middle or above	199 (80.2)	158 (79.8)	41 (82.0)		178 (79.1)	142 (78.9)	36 (80.0)	
unknown	3 (1.2)	1 (0.5)	2 (4.0)		3 (1.3)	3 (1.7)	0 (0.0)	

The Original sample refers to all participants initially included in the study. The Re-rating sample refers to participants with available video recordings of gait- and posture-related items (#3.9–#3.13) for re-evaluation. ^a^Education – unknown: three participants have unknown education; however, they have MMSE>24; therefore, they were included in our study. ^*^p: were estimated using one-way ANOVA test for continuous variables and the chi-squared test for categorical variables.

### 3.2 Model LOOCV performance and interpretation

**Figure 4:**
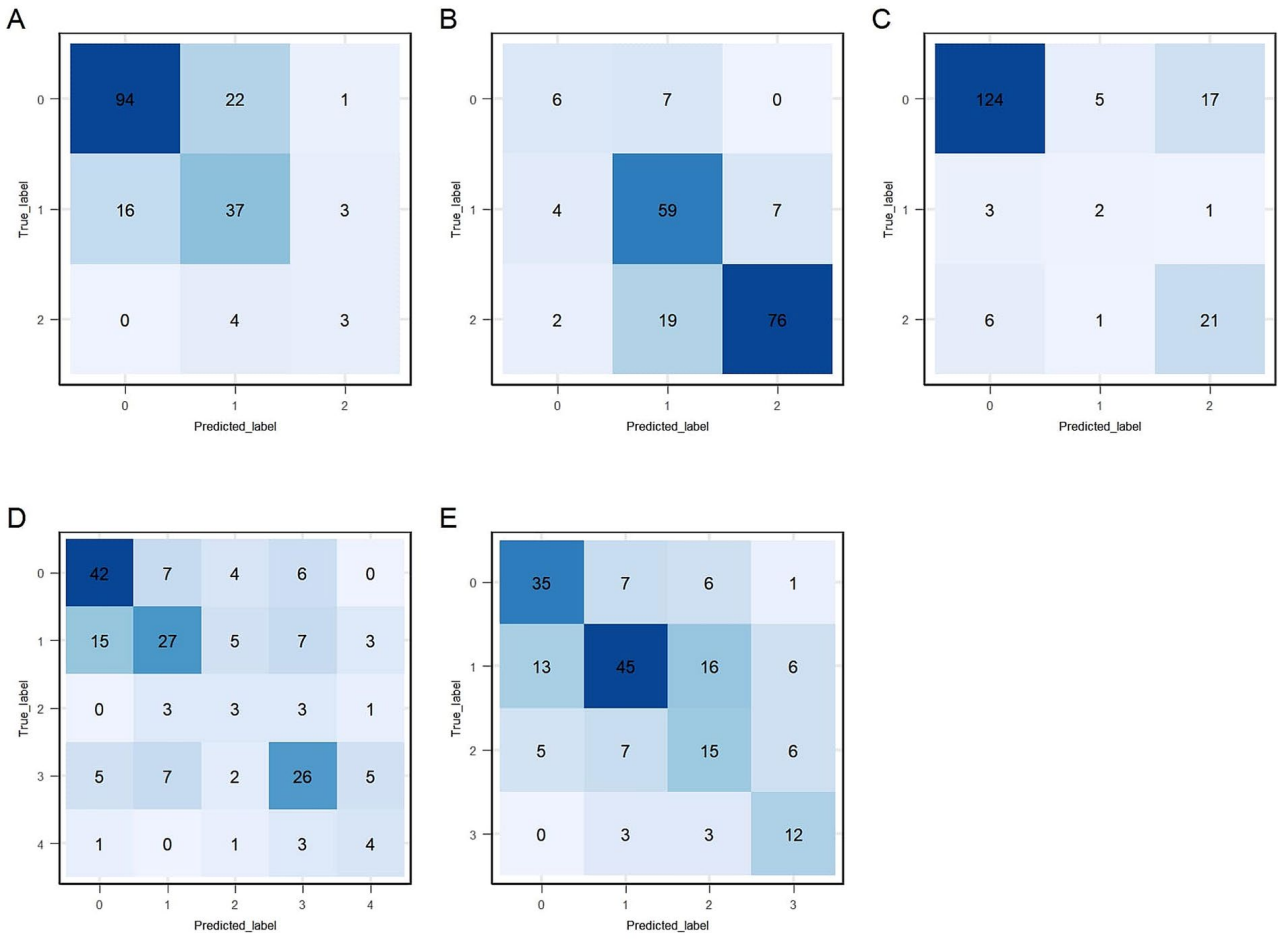
Confusion matrices for the training data. **(A–E)** Correspond to sections #3.9 to #3.13, respectively: #3.9 (arising from a chair), #3.10 (gait), #3.11 (freezing of gait), #3.12 (postural stability), and #3.13 (posture).

Predictive performance comparisons between XGBoost and SVM algorithms for each of the five gait and posture items based on LOOCV are presented in Table 2. XGBoost consistently demonstrated better performance compared to SVM; therefore, XGBoost was selected as the final predictive model for all five gait and posture items. Weighted F1 score of item #3.9 ~ #3.11 is above 0.7, while they are approximately 0.6 for item #3.12 and #3.13. Macro-F1 score of item #3.9 ~ #3.11 is above 0.6, while they are approximately 0.5 for item #3.12 and #3.13. Absolute accuracy of all five XGBoost models exceeded 55%, with items #3.9, #3.10, and #3.11 achieving values above 70%. Acceptable accuracy (within ±1 point of true scores) exceeded 80% for all models, with notably high accuracy (>90%) observed for items #3.9, and #3.10. *Kw* coefficients, indicating the level of agreement between true and predicted scores, were above 0.5, representing at least moderate agreement for all five models. Detailed precision, recall, and F1 values for each gait and posture item classification by XGBoost models are shown in Table 3. Overall, precision and recall varied across score categories and items, with lower score categories (indicating less severe impairment) generally showing higher classification performance, while higher score categories, particularly those with limited sample sizes, exhibited reduced performance. For item # 3.9, the model achieved strong classification performance for the unimpaired category (Score *0*), with LOOCV precision, recall, and F1 score of 0.855, 0.803, and 0.828, respectively. While performance for higher severity categories showed room for improvement, these categories were notably underrepresented (e.g., only 7 samples for Score *2/3/4* in LOOCV), which likely contributed to reduced model performance in those groups. In item # 3.10, the model demonstrated good performance for Score *2/3* with a precision of 0.916 and recall of 0.784 under LOOCV, while classification for Score *0* showed lower performance, with an F1 score of 0.48. The model also demonstrated robust performance for item # 3.11, achieving an F1 score of 0.889 for the non-impaired group (Score *0*) and 0.627 for higher severity categories (Score *2/3/4*) under LOOCV, suggesting reliable detection of both absence and presence of freezing phenomena, despite limited data for intermediate severity levels. Performance for items # 3.12 and # 3.13 followed similar trends, with higher classification metrics for lower severity categories and reduced performance for higher scores, primarily attributable to class imbalance and the small number of samples representing more severe symptoms. Confusion matrices for the training datasets of the final XGBoost model are presented in Figure 4.

**Table 2:** LOOCV and test performance of the models predicting scores on the five gait and posture items.

Type	Item, # (description)	Model	Weighted F1	ACC±0	ACC±1	*Kw*	*Macro F1*	fea_num^a^	*N*	Hyperparameters
LOOCV	3.9 (arising from chair)	XGBoost	0.748	74.4%	99.4%	0.52	0.626	35	180	gamma = 0.1, max_depth = 3, lambda = 3, eta = 0.1
		SVM	0.725	72.2%	98.9%	0.49	0.705	10	180	gamma = 0.1, cost = 1
	3.10 (gait)	XGBoost	0.786	78.3%	98.9%	0.64	0.695	35	180	gamma = 0.2, max_depth = 3, lambda = 5, eta = 0.05
		SVM	0.763	76.1%	100.0%	0.63	0.795	20	180	gamma = 0.01, cost = 0.1
	3.11 (freezing of gait)	XGBoost	0.828	81.7%	87.2%	0.53	0.6	30	180	gamma = 0.2, max_depth = 3, lambda = 4, eta = 0.1
		SVM	0.816	80.6%	88.9%	0.5	0.759	50	180	gamma = 0.01, cost = 1
	3.12 (postural stability)	XGBoost	0.571	56.7%	80.6%	0.51	0.481	30	180	gamma = 0.1, max_depth = 3, lambda = 3, eta = 0.05
		SVM	0.459	50.0%	76.7%	0.37	0.424	40	180	gamma = 0.1, cost = 1
	3.13 (posture)	XGBoost	0.6	59.4%	88.3%	0.5	0.572	40	180	gamma = 0.1, max_depth = 3, lambda = 5, eta = 0.1
		SVM	0.525	52.8%	93.9%	0.49	0.539	50	180	gamma = 0.01, cost = 1
TEST	3.9 (arising from chair)	XGBoost	0.767	77.8%	100.0%	0.573	0.783	35	45	gamma = 0.1, max_depth = 3, lambda = 3, eta = 0.1
	3.10 (gait)	XGBoost	0.715	71.1%	100.0%	0.539	0.679	35	45	gamma = 0.2, max_depth = 3, lambda = 5, eta = 0.05
	3.11 (freezing of gait)	XGBoost	0.777	82.2%	93.3%	0.664	0.823	30	45	gamma = 0.2, max_depth = 3, lambda = 4, eta = 0.1
	3.12 (postural stability)	XGBoost	0.408	40.0%	66.7%	0.278	0.422	30	45	gamma = 0.1, max_depth = 3, lambda = 3, eta = 0.05
	3.13 (posture)	XGBoost	0.463	46.7%	84.4%	0.308	0.397	40	45	gamma = 0.1, max_depth = 3, lambda = 5, eta = 0.1

^a^fea_num: the number of features that were included in constructing the models.

**Table 3:** LOOCV and test performance of the XGBoost models by class on predicting scores on the five gait and posture items.

Item, # (description)	Score categories	LOOCV				TEST			
		Precision	Recall	F1	*N* ^a^	Precision	Recall	F1	*N* ^a^
3.9 (arising from chair)	Class: 0 (Score *0*)	0.855	0.803	0.828	117	0.769	0.87	0.816	23
	Class: 1 (Score *1*)	0.587	0.661	0.622	56	0.789	0.714	0.75	21
	Class: 2 (Score *2/3/4*)	0.429	0.429	0.429	7	NA	0	NA	1
3.10 (gait)	Class: 0 (Score *0*)	0.5	0.462	0.48	13	0.75	0.75	0.75	4
	Class: 1 (Score *1*)	0.694	0.843	0.761	70	0.462	0.5	0.48	12
	Class: 2 (Score *2/3*)	0.916	0.784	0.844	97	0.821	0.793	0.807	29
3.11 (freezing of gait)	Class: 0 (Score *0*)	0.932	0.849	0.889	146	0.882	0.938	0.909	32
	Class: 1 (Score *1*)	0.25	0.333	0.286	6	NA	0	NA	5
	Class: 2 (Score *2/3/4*)	0.538	0.75	0.627	28	0.636	0.875	0.737	8
3.12 (postural stability)	Class: 0 (Score *0*)	0.667	0.712	0.689	59	0.562	0.6	0.581	15
	Class: 1 (Score *1*)	0.614	0.474	0.535	57	0.273	0.3	0.286	10
	Class: 2 (Score *2*)	0.2	0.3	0.24	10	0	0	NA	2
	Class: 3 (Score *3*)	0.578	0.578	0.578	45	0.462	0.353	0.4	17
	Class: 4 (Score *4*)	0.308	0.444	0.364	9	0	0	NA	1
3.13 (posture)	Class: 0 (Score *0*)	0.66	0.714	0.686	49	0.5	0.571	0.533	14
	Class: 1 (Score *1*)	0.726	0.562	0.634	80	0.526	0.526	0.526	19
	Class: 2 (Score *2*)	0.375	0.455	0.411	33	0.4	0.25	0.308	8
	Class: 3 (Score *3/4*)	0.48	0.667	0.558	18	0.2	0.25	0.222	4

^a^*N*: sample size.

**Figure 5:**
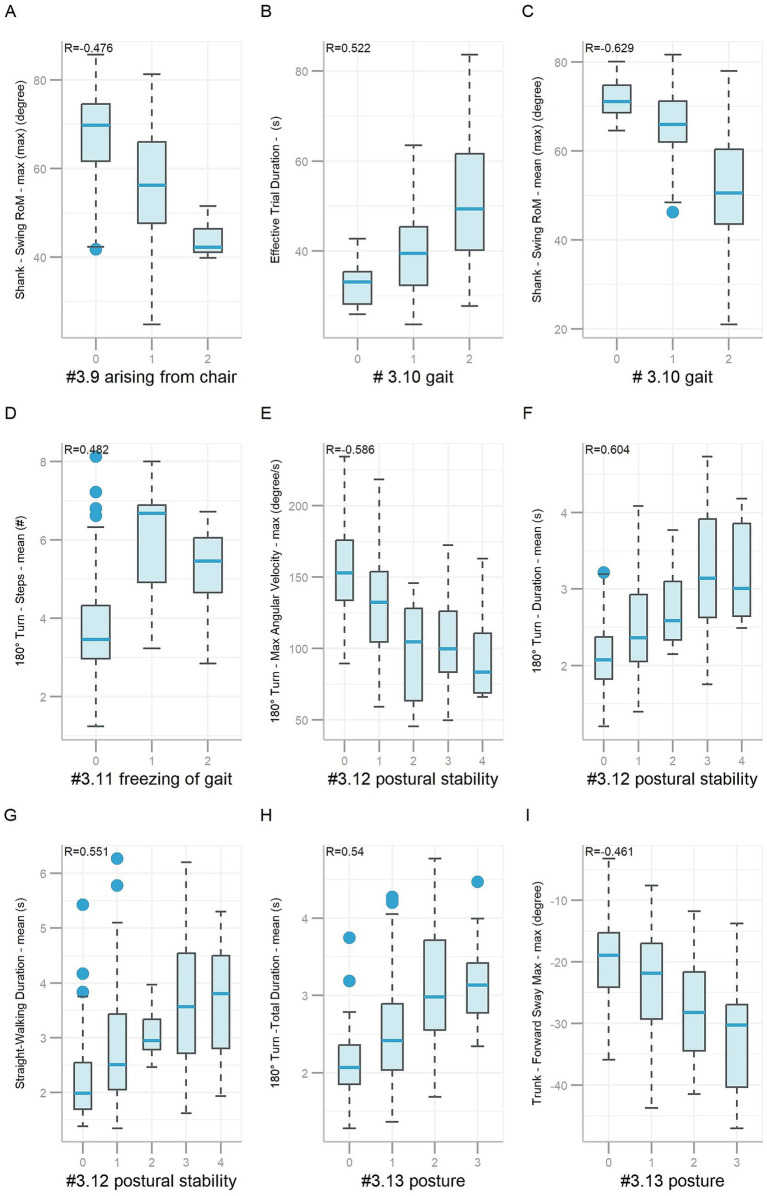
Boxplots of features based on gait and posture items #3.9 ~ #3.13. Nine boxplots labeled A to I, illustrating various metrics related to gait and posture in different scenarios. Each plot shows data for three or more class levels, with correlation coefficients (R values) indicated. Boxplot represents data as follows: the central line represents the median; the top and bottom lines of the box represent the 75th quantile (Q3) and 25th quantile (Q1), respectively; the top and bottom of the error bars indicate the “Maximum” (Q3+1.5*(Q3−Q1)) and “Minimum” (Q1−1.5*(Q3-Q1)), respectively; and dots represent outliers (outside the “Maximum” and “Minimum”).

Feature importance (Gain) utilized in the final predictive models for items #3.9 to #3.13 is detailed in Supplementary Tables 2–6. The total number of features selected for predictive models of items #3.9, #3.10, #3.11, #3.12, and #3.13 was 35, 35, 30, 30, and 40, respectively. Importantly, several key features demonstrated meaningful clinical correlations. For example, *Shank—Swing RoM—max (max)* had a negative correlation (*R* = −0.476, *p* < 0.001, Figure 5A) with scores on item #3.9. Higher scores on item #3.9 indicate more severe gait and posture impairment, which is consistent with reduced lower-limb mobility. A smaller shank swing range may reflect impaired lower-limb strength and coordination, affecting functional tasks such as standing up from a chair. *Effective Trial Duration* (R = 0.522, *p* < 0.001) had a positive correlation, while *Shank—Swing RoM—mean (max)* (*R* = −0.629, *p* < 0.001) had a negative correlation with scores on item #3.10 (Figures 5B,C), which were consistent with clinical findings. As the more severe the gait impairment is, the slower the walking speed will be and the smaller the range of motion of the shank will be. *180° Turn—Steps—mean* had a positive correlation (*R* = 0.482, *p* < 0.001, Figure 5D) with scores on item #3.11. Higher scores on item #3.11 indicate more severe gait and posture deficits, which is consistent with increased step count during turning. Increased mean step count during turning may indicate gait freezing tendencies or impaired postural control in PD patients. *180° Turn—Max Angular Velocity—max* (*R* = −0.586, *p* < 0.001) had a negative correlation, while *180° Turn—Duration—mean* (*R* = 0.604, *p* < 0.001) and *Straight-Walking Duration—mean* (*R* = 0.551, *p* < 0.001) had positive correlation with scores on item #3.12 (Figures 5E–G), which were consistent with clinical observations. As the more unsteady the participant is, the slower they walk, the more time they would spend on walking. *180° Turn -Total Duration—mean* had a positive correlation (*R* = 0.54, *p* < 0.001), while *Trunk—Forward Sway Max—max* had a negative correlation (*R* = −0.461, *p* < 0.001) with scores on item #3.13 (Figures 5H,I), which were also consistent with clinical findings. The more sever posture impairment, the more time the participant spends on turning. *Trunk—Forward Sway Max—max* was used to describe the sagittal projection of the trunk’s maximum forward tilt relative to the gravity vertical while walking (backward: positive value, forward: negative value). The larger the absolute value of the negative value, the greater the participant’s trunk forward angle while walking, the severer the posture damage, the higher scores on item #3.13.

Predicting the MDS-UPDRS III gait and posture subscale score via LOOCV on the training data achieved a MAE of 1.349 and a RMSE of 1.645. The correlation between predicted and actual subscale scores was strong (R = 0.798).

### 3.3 Sensor contributions

Sensor contributions to predictive models for each gait and posture item are presented in Supplementary Table 7. Because some features incorporated multiple sensors, the cumulative sensor contributions exceeded 100% for some gait and posture items. Shank sensors provided the greatest contributions for gait and posture items #3.10 (48.6%), #3.11 (43.3%), #3.12 (43.3%), and #3.13 (35%). For item #3.9, the chest sensor contributed most (37.1%), followed by the shank sensor (31.4%) and lumbar (28.6%). Comparison of the contributions of different sensors revealed that shank sensors provided the greatest contribution, followed by chest sensor.

### 3.4 Independent clinical evaluation

**Figure 6:**
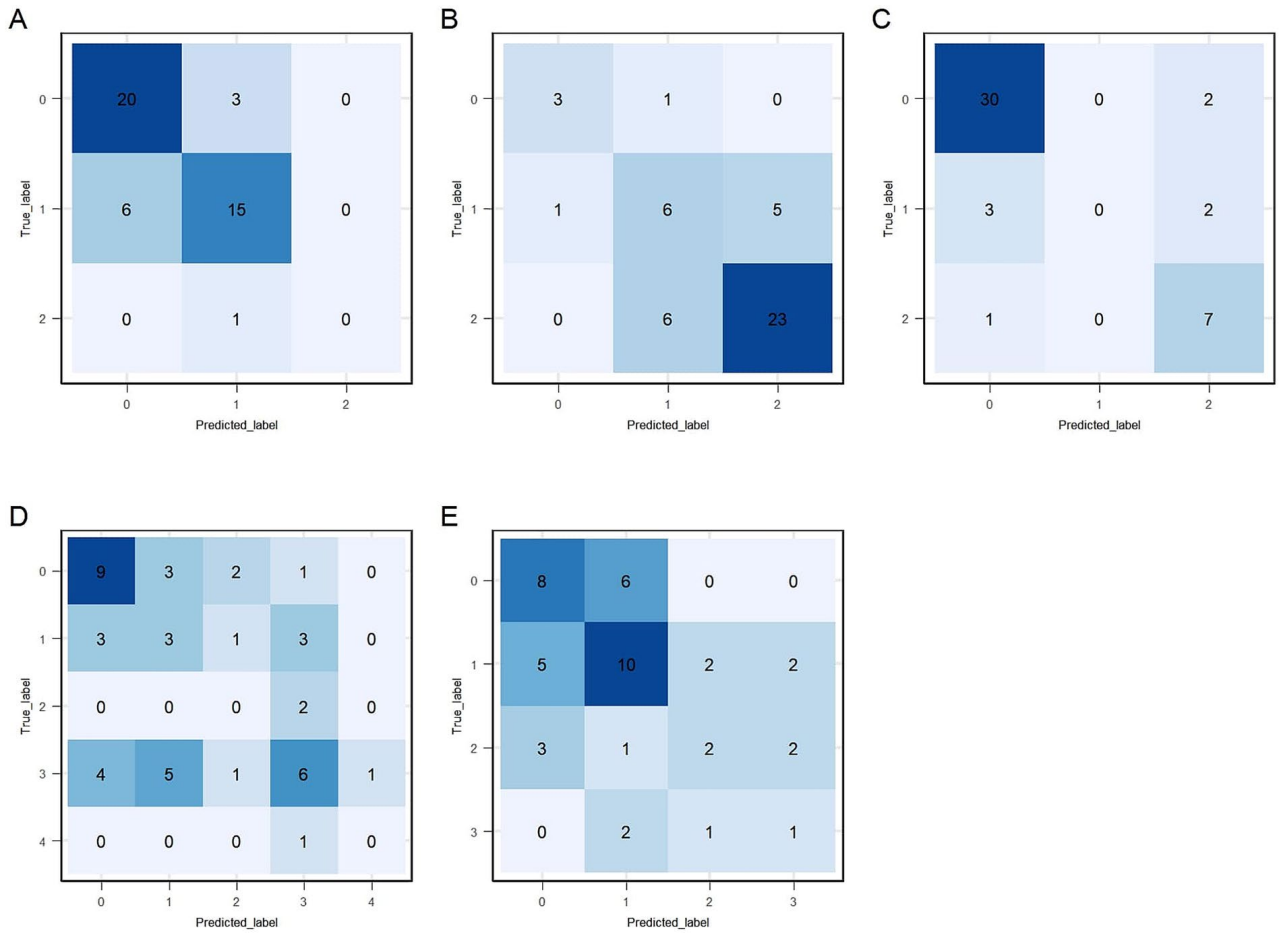
Confusion matrices for the test data. **(A–E)** Correspond to sections #3.9 to #3.13, respectively: #3.9 (arising from a chair), #3.10 (gait), #3.11 (freezing of gait), #3.12 (postural stability), and #3.13 (posture).

The full dataset of 225 participants was divided into training (80%) and independent test sets (20%). Performance evaluations of the final predictive models on the independent test set are provided in Tables 2, 3. Test data performance closely matched training LOOCV results, confirming the models’ reproducibility capability. Weighted F1, macro-F1, absolute, and acceptable accuracy values for items #3.9, #3.10, and #3.11 remained high (above 0.7, 0.67, 70, and 90%, respectively). However, predictive performance for items #3.12 and #3.13 was suboptimal. Table 3 shows class-specific evaluations on test data. The predictive model for item #3.9 showed high precision and recall for Score *0* and Score *1* (above 70%). No participants were predicted to be Score *2/3/4*, which lead to the precision value to be NA. Model on gait and posture item #3.10 had high precision and recall value on Score *0* and Score *2/3* (above 75%). The fraction of instances correctly classified as Score *1* out of all instances the model predicted to belong to Score *1* was 46.2% (precision), while it was 50% for recall. Model on gait and posture item #3.11 exhibited good performance for Score *0* and Score *2/3/4* but limited ability for Scores *1*. Confusion matrices for the test dataset of the final XGBoost model are presented in Figure 6. Furthermore, the predictive model for gait and posture subscale was evaluated on independent test data, which achieved a MAE of 1.432, a RMSE of 1.776, and a strong correlation coefficient (R = 0.818) between the predicted gait and posture subscale and true gait and posture subscale.

## 4 Discussion

This study developed five predictive models using wearable sensor-based kinematic features to assess the severity of gait and posture symptoms in PD, as measured by five MDS-UPDRS III gait and posture items (#3.9–# 3.13). Our findings demonstrate the potential of wearable sensor-based gait analysis as a quantitative, automated, and standardized assessment tool, addressing the limitations of conventional clinician-rated evaluations. In our study, XGBoost consistently outperformed SVM across all five gait and posture items. Notably, all five XGBoost predictive models demonstrated acceptable performance, achieving acceptable accuracy values exceeding 80%, with items #3.9, #3.10, and #3.11 surpassing 70% absolute accuracy during LOOCV on the training data. Consistently, the weighted F1 scores for these items were above 0.70 and the macro-F1 scores were above 0.60, reflecting reliable model performance in the context of class imbalance and multi-class symptom classification. Additionally, the performance of these models on independent test data closely matched the LOOCV outcomes, highlighting the robust generalizability of our predictive models.

Recent studies have highlighted the clinical value of gait-derived features in PD. Cao et al. (2020) reported that step-to-step sequence effect is associated with freezing of gait and can be mitigated by visual cues. Park et al. (2025) used machine learning on gait parameters to classify neurological disorders, while Taximaimaiti and Wang (2021) found FOG linked to more severe motor and non-motor symptoms. Several prior studies have further explored sensor-based methods for PD gait and posture symptom assessment. Abujrida et al. (2020) used smartphone-derived gait features to predict MDS-UPDRS II item scores (#2.12 walking and balance, #2.13 freezing), while Safarpour et al. (2022) employed wearable sensors to predict PIGD scores, achieving a moderate correlation (0.61). However, these studies either focused on limited clinical items or lacked a standardized laboratory assessment protocol. In contrast, our study is the first to simultaneously predict all five MDS-UPDRS III gait and posture items with acceptable accuracy (>80%) using a single, structured gait assessment. This methodological advancement underscores the feasibility of wearable sensor-based monitoring as a reliable alternative to subjective clinical evaluations.

Given the clinical significance of gait and posture symptoms in PD, we further analyzed how specific kinematic features correlated with them to better understand the observed results. Postural instability is a significant symptom of PD (Kim et al., 2013). The more severe symptoms that PD patients have, the more they struggle to maintain their balance, which reduces walking speed. The results showed that the feature *180° Turn—Max Angular Velocity—max* was moderately negatively correlated (*R* = −0.586, *p* < 0.001, Supplementary Table 5) with scores on item #3.12 (postural stability). Higher scores on item #3.12 resulted in longer turning durations of these PD patients on the assessment. In addition, PD patients with more severe posture impairment tended to have symptoms such as more severe flexion, scoliosis, or leaning to one side. Our result shows that feature *180° Turn—Trunk—Sagittal Mean Sway—mean*, which was used to measure of the sagittal projection of the torso’s tilt relative to the gravity vertical through turning process, was moderately negatively correlated (*R* = −0.521, *p* < 0.001, Supplementary Table 6) with scores on item #3.13 (posture). Moreover, our result showed that *180° Turn—Duration—mean* (*R* = 0.604, *p* < 0.001, Supplementary Table 5) had positive relationship, while *180° Turn—Max Angular Velocity—max* (*R* = −0.586, *p* < 0.001, Supplementary Table 5) had negative relationship with scores on item #3.12, reinforcing findings by Ahmed et al. (2017), who reported that impaired postural stability was associated with specific gait parameters, such as reduced walking speed. Therefore, our results were consistent with the known clinical manifestations of PD on these gait and posture symptom domains.

Beyond gait and posture symptoms, our findings also revealed strong correlations between gait parameters and bradykinesia severity, measured by the MDS-UPDRS III bradykinesia subscale (sum of items 3.2, 3.4, 3.5, 3.6, 3.7, 3.8, and 3.14) (Zhu et al., 2024). For example, *180° Turn—Mean Angular Velocity—mean* (*R* = −0.47, *p* < 0.001) and *Shank—Swing RoM—mean (max)* (*R* = −0.41, *p* < 0.001) correlated negatively with bradykinesia subscale, suggesting that slower turning speeds and reduced shank motion reflect movement slowness, a key characteristic of PD-related bradykinesia.

To optimize predictive accuracy, we compared two widely used machine learning algorithms: support vector machine (SVM) and extreme gradient boosting (XGBoost). SVM identifies an optimal separating hyperplane by maximizing the margin between classes. To capture complex, non-linear relationships, SVM employs kernel functions—such as the RBF—which map the original low-dimensional data into a higher-dimensional feature space, increasing the likelihood that otherwise inseparable patterns become linearly separable (Noble, 2006). However, SVM performance is sensitive to kernel selection, and suboptimal kernel choices may limit its classification effectiveness. In contrast, XGBoost, an ensemble learning approach based on gradient boosting, is particularly well-suited for modeling complex, non-linear feature interactions. It integrates effective built-in feature selection through the Gain metric, where higher Gain values reflect greater feature importance (Burnwal and Jaiswal, 2023). In addition, XGBoost incorporates L1 and L2 regularization to mitigate overfitting and improve model generalization. Although XGBoost offers robust predictive performance, its computational demands and sensitivity to hyperparameter tuning must be carefully managed. Our findings demonstrated that XGBoost consistently outperformed SVM under LOOCV, likely due to its superior capacity for capturing non-linear gait kinematic patterns and effectively identifying relevant features.

This study has several considerations. First, a limited sample size required merging categories with fewer participants, potentially influencing differentiation among severity levels. Future studies with larger and more diverse participant groups could improve the model’s accuracy and reliability. Second, all data in this study were obtained from a single clinical site, and no external datasets were used for model validation. This limitation may affect the generalizability of our findings. Independent validation using data from other clinical settings will be important to further establish the model’s applicability. Third, the initial clinical scores in our study were provided by a single movement disorder specialist, which may introduce subjectivity, a common limitation in clinical practice. To address this concern and enhance the reliability of the ground truth labels, we conducted an additional multi-rater, multi-round re-rating process based on video recordings of the gait and posture-related items (MDS-UPDRS III #3.9–#3.13). This procedure helped reduce potential bias and improve the robustness of the reference standards used for model development. Finally, as the assessments were conducted in a laboratory setting, future studies could benefit from evaluating wearable sensor-based methods in home-based, naturalistic environments. This approach could enable remote monitoring of gait and posture symptoms, supporting timely interventions and improving patient outcomes.

## 5 Conclusion

This study demonstrates the feasibility of wearable sensor-based gait analysis for predicting MDS-UPDRS III gait and posture scores in PD patients, which reinforcing the potential of objective, sensor-based PD assessment tools.

## Data Availability

The original contributions presented in the study are included in the article/Supplementary material, further inquiries can be directed to the corresponding authors.

## Glossary

PD: Parkinson’s disease
MDS-UPDRS: Movement Disorder Society’s Unified Parkinson’s Disease Rating Scale
PIGD: Postural Instability Gait Difficulty
MMSE: Mini-Mental State Examination
CE Medical: ConformitÈ Europëenne Medical
NMPA: National Medical Products Administration
FDA: U.S. Food and Drug Administration
IMU: inertial measurement unit
SW-1: straight-walk I
T1: 180° Turn I
IC: initial contact
TC: terminal contact
LOOCV: leave-one-out cross-validation
XGBoost: extreme gradient boosting
LASSO: least absolute shrinkage and selection operator
ACC ± 0: absolute accuracy
ACC ± 1: acceptable accuracy
Kw: Cohen’s weighted kappa
MAE: mean absolute error
RMSE: root mean square error
R: Spearman correlation coefficient
