# Supplementary material for: Objective assessment of gait and posture symptoms in Parkinson’s disease using wearable sensors and machine learning
Source: Front Aging Neurosci. 2025 Aug 8;17:1618764. doi: 10.3389/fnagi.2025.1618764 (PMC12370646; doi:10.3389/fnagi.2025.1618764)
Supplement: Supplementary file 1 [file Data_Sheet_1.docx]

Supplementary Materials for
Objective Assessment of Gait and Posture Symptoms in Parkinson’s Disease Using Wearable Sensors and Machine Learning

# Supplementary Figures and Tables

This file includes 7 tables:
eTable 1 Feature definition
eTable 2 Features for model construction on item # 3.9 (arising from chair)
eTable 3 Features for model construction on item # 3.10 (gait)
eTable 4 Features for model construction on item #3.11 (freezing of gait)
eTable 5 Features for model construction on item # 3.12 (postural stability)
eTable 6 Features for model construction on item # 3.13 (posture)
eTable 7 Contribution of different sensors to each gait and posture item model

**eTable 1 Feature definition**

| **Part 1 Segmentation-gait cycle-based features** |  |
| --- | --- |
| Step Length R | A measurement of Right Step Length is defined as the straight line distance from the left heel to the right heel in a Left Gait Cycle at the time when the right heel strikes ground. Finally, the average is taken across all gait cycles within a straight-walk section. |
| Step Length R SD | Standard Deviation of all the measurements of Right Step Length through all the gait cycles within a straight-walk section. |
| Step Length L | A measurement of Left Step Length is defined as the straight line distance from the right heel to the left heel in a Right Gait Cycle at the time when the left heel strikes ground. Finally, the average is taken across all gait cycles within a straight-walk section. |
| Step Length L SD | Standard Deviation of all the measurements of Left Step Length through all the gait cycles within a straight-walk section. |
| Step Length | Mean of the measurements of Left Step Length and Right Step Length in each gait cycle. Finally, the average is taken across all gait cycles within a straight-walk section. |
| Step Length SD | Standard Deviation of all the measurements of Left Step Length and Right Step Length through all the gait cycles within a straight-walk section. |
| Gait Speed R | A measurement of Right Gait Speed is defined as the ratio of a measurement of Right Stride Length to its corresponding Right Gait Cycle’s duration. Finally, the average is taken across all gait cycles within a straight-walk section. |
| Gait Speed R SD | Standard Deviation of all the measurements of Right Gait Speed through all the gait cycles within a straight-walk section. |
| Gait Speed L | A measurement of Left Gait Speed is defined as the ratio of a measurement of Left Stride Length to its corresponding Left Gait Cycle’s duration. Finally, the average is taken across all gait cycles within a straight-walk section. |
| Gait Speed L SD | Standard Deviation of all the measurements of Left Gait Speed through all the gait cycles within a straight-walk section. |
| Gait Speed | Mean of the measurements of Left Gait Speed and Right Gait Speed in each gait cycle. Finally, the average is taken across all gait cycles within a straight-walk section. |
| Gait Speed SD | Standard Deviation of all the measurements of Left and Right Gait Speed through all the gait cycles within a straight-walk section. |
| Stride Length R | The distance in the direction of progression between two consecutive ground contact points of the right foot within the right gait cycle. Finally, the average is taken across all gait cycles within a straight-walk section. |
| Stride Length R SD | Standard Deviation of all the measurements of Right Stride Length through all the gait cycles within a straight-walk section. |
| Stride Length L | The distance in the direction of progression between two consecutive ground contact points of the left foot within the left gait cycle. Finally, the average is taken across all gait cycles within a straight-walk section. |
| Stride Length L SD | Standard Deviation of all the measurements of Left Stride Length through all the gait cycles within a straight-walk section. |
| Stride Length | Mean of the measurements of Left Stride Length and Right Stride Length in each gait cycle. Finally, the average is taken across all gait cycles within a straight-walk section. |
| Stride Length SD | Standard Deviation of all the measurements of Left Stride Length and Right Stride Length through all the gait cycles within a straight-walk section. |
| Gait Cycle R | A measurement of Right Gait Cycle is defined as the time interval between two successive events that the right heel strikes ground. Finally, the average is taken across all gait cycles within a straight-walk section. |
| Gait Cycle R SD | Standard Deviation of all the measurements of Right Gait Cycle through all the gait cycles within a straight-walk section. |
| Gait Cycle L | A measurement of Left Gait Cycle is defined as the time interval between two successive events that the Left heel strikes ground. Finally, the average is taken across all gait cycles within a straight-walk section. |
| Gait Cycle L SD | Standard Deviation of all the measurements of Leftt Gait Cycle through all the gait cycles within a straight-walk section. |
| Gait Cycle | Mean of the measurements of Left Gait Cycle and Right Gait Cycle in each gait cycle. Finally, the average is taken across all gait cycles within a straight-walk section. |
| Gait Cycle SD | Standard Deviation of all the measurements of Left Gait Cycle and Right Gait Cycle through all the gait cycles within a straight-walk section. |
| Cadence R | A measurement of Right Cadence is defined as the inverse of the time interval between a left-heel-striking and the successive right-heel-striking. (unit: step/min) . Finally, the average is taken across all gait cycles within a straight-walk section. |
| Cadence R SD | Standard Deviation of all the measurements of Right Cadence hrough all the gait cycles within a straight-walk section. |
| Cadence L | A measurement of Left Cadence is defined as the inverse of the time interval between a right-heel-striking and the successive left-heel-striking. (unit: step/min). Finally, the average is taken across all gait cycles within a straight-walk section. |
| Cadence L SD | Standard Deviation of all the measurements of Left Cadence through all the gait cycles within a straight-walk section. |
| Cadence | Mean of the measurements of Left Cadence and Right Cadence in each gait cycle. Finally, the average is taken across all gait cycles within a straight-walk section. |
| Cadence SD | Standard Deviation of all the measurements of Left Cadence and Right Cadence through all the gait cycles within a straight-walk section. |
| Double Support R | A measurement of Right Double Support is defined as the ratio of the time length, during which both limbs keep contacting with the ground, to its corresponding Right Gait Cycle. Finally, the average is taken across all gait cycles within a straight-walk section. |
| Double Support R SD | Standard Deviation of all the measurements of Right Double Support through all the gait cycles within a straight-walk section. |
| Double Support L | A measurement of Left Double Support is defined as the ratio of the time length, during which both limbs keep contacting with the ground, to its corresponding Left Gait Cycle. Finally, the average is taken across all gait cycles within a straight-walk section. |
| Double Support L SD | Standard Deviation of all the measurements of Left Double Support through all the gait cycles within a straight-walk section. |
| Double Support | Mean of the measurements of Left Double Support and Right Double Support in each gait cycle. Finally, the average is taken across all gait cycles within a straight-walk section. |
| Double Support SD | Standard Deviation of all the measurements of Left Double Support and Right Double Support through all the gait cycles within a straight-walk section. |
| Swing R | A measurement of Right Swing is defined as the ratio of the period, during which the right limb is unconnected with the ground, to its corresponding Right Gait Cycle. Finally, the average is taken across all gait cycles within a straight-walk section. |
| Swing R SD | Standard Deviation of all the measurements of Right swing through all the gait cycles within a straight-walk section . |
| Swing L | A measurement of Left Swing is defined as the ratio of the period, during which the Left limb is unconnected with the ground, to its corresponding Left Gait Cycle. Finally, the average is taken across all gait cycles within a straight-walk section. |
| Swing L SD | Standard Deviation of all the measurements of Left swing through all the gait cycles within a straight-walk section. |
| Swing | Mean of the measurements of Left Swing and Right Swing in each gait cycle. Finally, the average is taken across all gait cycles within a straight-walk section. |
| Swing SD | Standard Deviation of all the measurements of Left Swing and Right Swing through all the gait cycles within a straight-walk section. |
| Stance R | A measurement of Right Stance is defined as the ratio of the period, during which the right limb is touching the ground, to its corresponding Right Gait Cycle. Finally, the average is taken across all gait cycles within a straight-walk section. |
| Stance R SD | Standard Deviation of all the measurements of Right Stance through all the gait cycles within a straight-walk section. |
| Stance L | A measurement of Left Stance is defined as the ratio of the period, during which the Left limb is touching the ground, to its corresponding Left Gait Cycle. Finally, the average is taken across all gait cycles within a straight-walk section. |
| Stance L SD | Standard Deviation of all the measurements of Left Stance through all the gait cycles within a straight-walk section. |
| Stance | Mean of the measurements of Left Stance and Right Stance in each gait cycle. Finally, the average is taken across all gait cycles within a straight-walk section. |
| Stance SD | Standard Deviation of all the measurements of Left Stance and Right Stance through all the gait cycles within a straight-walk section. |
| Shank - Forward Swing Max R | The maximum angle of the forward swing of the right shank within each gait cycle. Finally, the average is taken across all gait cycles within a straight-walk section. |
| Shank - Forward Swing Max R SD | Standard deviation of of all maximum forward swing angles of the right shank through all the gait cycles within a straight-walk section. |
| Shank - Forward Swing Max L | The maximum angle of the forward swing of the Left shank within each gait cycle. Finally, the average is taken across all gait cycles within a straight-walk section. |
| Shank - Forward Swing Max L SD | Standard deviation of all maximum forward swing angles of the Left shank through all the gait cycles within a straight-walk section. |
| Shank - Forward Swing Max | Mean of Left Shank - Forward Swing Max and Right Shank - Forward Swing Max in each gait cycle. Finally, the average is taken across all gait cycles within a straight-walk section. |
| Shank - Forward Swing Max SD | Standard Deviation of all the measurements of Left Shank - Forward Swing Max and Right Shank - Forward Swing Max through all the gait cycles within a straight-walk section. |
| Shank - Backward Swing Max R | The maximum angle of the backward swing of the right shank within each gait cycle. Finally, the average is taken across all gait cycles within a straight-walk section. |
| Shank - Backward Swing Max R SD | Standard deviation of of all maximum backward swing angles of the right shank through all the gait cycles within a straight-walk section. |
| Shank - Backward Swing Max L | The maximum angle of the backward swing of the Left shank within each gait cycle. Finally, the average is taken across all gait cycles within a straight-walk section. |
| Shank - Backward Swing Max L SD | Standard deviation of of all maximum backward swing angles of the Left shank through all the gait cycles within a straight-walk section . |
| Shank - Backward Swing Max | Mean of Left Shank - Backward Swing Max and Right Shank - Backward Swing Max in each gait cycle. Finally, the average is taken across all gait cycles within a straight-walk section. |
| Shank - Backward Swing Max SD | Standard Deviation of all the measurements of Left Shank - Backward Swing Max and Right Shank - Backward Swing Max through all the gait cycles within a straight-walk section. |
| Shank - Max Sagittal Angular Velocity R | The maximum angular velocity values of the right shank’s swings within each gait cycle. Finally, the average is taken across all gait cycles within a straight-walk section. |
| Shank - Max Sagittal Angular Velocity R SD | Standard deviation of all the maximum angular velocity values of the right shank’s swings through all the gait cycles within a straight-walk section. |
| Shank - Max Sagittal Angular Velocity L | The maximum angular velocity values of the Left shank’s swings within each gait cycle. Finally, the average is taken across all gait cycles within a straight-walk section. |
| Shank - Max Sagittal Angular Velocity L SD | Standard deviation of all the maximum angular velocity values of the Left shank’s swings through all the gait cycles within a straight-walk section . |
| Shank - Max Sagittal Angular Velocity | Mean of Left Shank - Max Sagittal Angular Velocity and Right Shank - Max Sagittal Angular Velocity in each gait cycle. Finally, the average is taken across all gait cycles within a straight-walk section. |
| Shank - Max Sagittal Angular Velocity SD | Standard Deviation of all the measurements of left Shank - Max Sagittal Angular Velocity and Right Shank - Max Sagittal Angular Velocity through all the gait cycles within a straight-walk section. |
| Shank - Swing Speed R | The average swing velocity of the right foot during the swing phase in each gait cycle. Finally, the average is taken across all gait cycles within a straight-walk section. |
| Shank - Swing Speed R SD | Standard Deviation of all the measurements of Shank- Right Swing Speed through all the gait cycles within a straight-walk section. |
| Shank - Swing Speed L | The average swing velocity of the left foot during the swing phase in each gait cycle. Finally, the average is taken across all gait cycles within a straight-walk section. |
| Shank - Swing Speed L SD | Standard Deviation of all the measurements of Shank-Left Swing Speed through all the gait cycles within a straight-walk section. |
| Shank - Swing Speed | Mean of the measurements of Shank-Left Swing Speed and Shank-Right Swing Speed in each gait cycle. Finally, the average is taken across all gait cycles within a straight-walk section. |
| Shank - Swing Speed SD | Standard Deviation of all the measurements of Shank-Left Swing Speed and Shank-Right Swing Speed through all the gait cycles within a straight-walk section. |
| Stride Velocity Asymmetry | Among the pairs of successive Left Gait Cycles and Right Gait Cycles in a straight-walk section, a measurement of Stride Velocity Asymmetry is defined as the ratio of the absolute value of the difference, between the successive Left Stride Velocity and Right Stride Velocity, to the larger one of them. Finally, the average is taken across all gait cycles within a straight-walk section. |
| Stride Velocity Asymmetry SD | Standard Deviation of all the measurements of Stride Velocity Asymmetry through all the gait cycles within a straight-walk section. |
| Stride Velocity Difference | Among the pairs of successive Left Gait Cycles and Right Gait Cycles in a straight-walk section, a measurement of Stride Velocity Absolute Deviation is defined as the absolute value of the difference between the successive Left Stride Velocity and Right Stride Velocity. Finally, the average is taken across all gait cycles within a straight-walk section. |
| Stride Velocity Difference SD | Standard Deviation of all the measurements of Stride Velocity Difference through all the gait cycles within a straight-walk section. |
| Stride Length Asymmetry | Among the pairs of successive Left Gait Cycles and Right Gait Cycles in a straight-walk section, the parameter is calculated as the ratios of the absolute values of the differences between the successive Left Stride Lengths and Right Stride Lengths to the larger ones of them. Finally, the average is taken across all gait cycles within a straight-walk section. |
| Stride Length Asymmetry SD | Standard Deviation of all the measurements of Stride Length Asymmetry through all the gait cycles within a straight-walk section. |
| Stride Length Difference | Among the pairs of successive Left Gait Cycles and Right Gait Cycles in a straight-walk section, the parameter is defined as the absolute values of the differences between the successive Left Stride Lengths and Right Stride Lengths. Finally, the average is taken across all gait cycles within a straight-walk section. |
| Stride Length Difference SD | Standard Deviation of all the measurements of Stride Length Difference through all the gait cycles within a straight-walk section. |
| Swing Asymmetry | Among the pairs of successive Left Gait Cycles and Right Gait Cycles in a straight-walk section, the parameter is calculated as the ratios of the absolute values of the differences between the successive Left Swings and Right Swings, to the larger ones of them. Finally, the average is taken across all gait cycles within a straight-walk section. |
| Swing Asymmetry SD | Standard Deviation of all the measurements of Swing Asymmetry through all the gait cycles within a straight-walk section. |
| Swing Absolute Difference | Among the pairs of successive Left Gait Cycles and Right Gait Cycles in a straight-walk section, the parameter is calculated as the absolute values of the differences between the successive Left Swings and Right Swings. Finally, the average is taken across all gait cycles within a straight-walk section. |
| Swing Absolute Difference SD | Standard Deviation of all the measurements of Swing Absolute Difference through all the gait cycles within a straight-walk section. |
| Stance Asymmetry | Among the pairs of successive Left Gait Cycles and Right Gait Cycles in a straight-walk section, the parameter is calculated as the ratios of the absolute values of the differences between the successive Left Stances and Right Stances, to the larger ones of them. Finally, the average is taken across all gait cycles within a straight-walk section. |
| Stance Asymmetry SD | Standard Deviation of all the measurements of Stance Asymmetry through all the gait cycles within a straight-walk section. |
| Stance Absolute Difference | Among the pairs of successive Left Gait Cycles and Right Gait Cycles in a straight-walk section, the parameter is calculated as the absolute values of the differences between the successive Left Stances and Right Stances. Finally, the average is taken across all gait cycles within a straight-walk section. |
| Stance Absolute Difference SD | Standard Deviation of all the measurements of Stance Absolute Difference through all the gait cycles within a straight-walk section. |
| Shank - RoM Asymmetry | Among the pairs of successive Left Gait Cycles and Right Gait Cycles in a straight-walk section, the parameter is calculated as the ratios of the absolute values of the differences between the successive left shank’s range and the right shank’s range., to the larger ones of them. Finally, the average is taken across all gait cycles within a straight-walk section. |
| Shank - RoM Asymmetry SD | Standard Deviation of all the measurements of Shank - RoM Asymmetry through all the gait cycles within a straight-walk section. |
| Shank - RoM Absolute Difference | Among the pairs of successive Left Gait Cycles and Right Gait Cycles in a straight-walk section, the parameter is calculated as the absolute values of the differences between the successive left shank’s range and the right shank’s range. Finally, the average is taken across all gait cycles within a straight-walk section. |
| Shank - RoM Absolute Difference SD | Standard Deviation of all the measurements of Shank - RoM Absolute Difference through all the gait cycles within a straight-walk section. |
| Shank - Asymmetry of Max Sagittal Angular Velocity | Among the pairs of successive Left Gait Cycles and Right Gait Cycles in a straight-walk section, the parameter is calculated as the ratios of the absolute values of the differences between the successive left shank’s Max Sagittal Angular Velocity and the right shank’s Max Sagittal Angular Velocity, to the larger ones of them. Finally, the average is taken across all gait cycles within a straight-walk section. |
| Shank - Asymmetry of Max Sagittal Angular Velocity SD | Standard Deviation of all the measurements of Shank - Asymmetry of Max Sagittal Angular Velocity through all the gait cycles within a straight-walk section. |
| Shank - Difference of Max Sagittal Angular Velocity | Among the pairs of successive Left Gait Cycles and Right Gait Cycles in a straight-walk section, the parameter is calculated as the absolute values of the differences between the successive left shank’s Max Sagittal Angular Velocity and the right shank’s Max Sagittal Angular Velocity. Finally, the average is taken across all gait cycles within a straight-walk section. |
| Shank - Difference of Max Sagittal Angular Velocity SD | Standard Deviation of all the measurements of Shank - Difference of Max Sagittal Angular Velocity through all the gait cycles within a straight-walk section. |
| Shank - Symbolic Symmetry Index | Can be applied to describe the symmetry of shanks’ movements in a straight-walk section. In each gait cycle, a measurement of Shank Symbolic Symmetry Index is performed, and finally, the average is taken across all gait cycles within a straight-walk section. An excellent performance means that the parameter approaches zero, while on the contrary, it approaches 100%. |
| Shank - Symbolic Symmetry Index SD | Standard Deviation of all the measurements of Shank - Symbolic Symmetry Index through all the gait cycles within a straight-walk section. |
| Mean Phase Difference | The relative mean phase difference reflects the coordination between the left and right foot during walking. From a computational perspective, it also represents the degree to which the phase value in each gait cycle deviates from 180 degrees, indicating the precision of phase generation. Finally, the average is taken across all gait cycles within a straight-walk section. An excellent performance means that the parameter approaches zero, while on the contrary, it approaches 100%. |
| Phase Coordination Index | The calculation method is the percentage ratio of the standard deviation of all relative gait cycle-based phase differences to the mean of all relative phase differences, combined with the relative Mean Phase Difference. |
| Coordination | Among the pairs of successive Left Gait Cycles and Right Gait Cycles in a straight-walk section, the parameter is calculated as the ratios of the absolute values of the differences between the successive left Step Length and the right Step Length, to the mean value of left Step Length and the right Step Length. Finally, the average is taken across all gait cycles within a straight-walk section. |
| Coordination SD | Standard Deviation of all the measurements of Coordination through all the gait cycles within a straight-walk section. |
| Stride Variability | The variability of stride length within the gait cycle during walking. Finally, the average is taken across all gait cycles within a straight-walk section. |
| Trunk - Max Coronal Angular Velocity | Mean of the measurements of the coronal projection of the torso’s maximum angular velocity across all gait cycles within a straight-walk section. |
| Trunk - Max Coronal Angular Velocity SD | Standard Deviation of the measurements of the coronal projection of the torso’s maximum angular velocity across all gait cycles within a straight-walk section. |
| Trunk - Right Sway Max | Mean of the measurements of the coronal projection of the torso’s maximum right tilt relative to the gravity vertical across all gait cycles within a straight-walk section. |
| Trunk - Right Sway Max SD | Standard Deviation of the measurements of the coronal projection of the torso’s maximum right tilt relative to the gravity vertical through all the gait cycles within a straight-walk section. |
| Trunk - Left Sway Max | Mean of the measurements of the coronal projection of the torso’s maximum left tilt relative to the gravity vertical across all gait cycles within a straight-walk section. |
| Trunk - Left Sway Max SD | Standard Deviation of the measurements of the coronal projection of the torso’s maximum left tilt relative to the gravity vertical through all the gait cycles within a straight-walk section. |
| Trunk - Max Sagittal Angular Velocity | Mean of the measurements of the sagittal projection of the torso’s maximum angular velocity across all gait cycles within a straight-walk section. |
| Trunk - Max Sagittal Angular Velocity SD | Standard Deviation of the measurements of the sagittal projection of the torso’s sagittal maximum angular velocity across all gait cycles within a straight-walk section. |
| Trunk - Backward Sway Max | Mean of the measurements of the sagittal projection of the torso’s maximum backward tilt relative to the gravity vertical across all gait cycles within a straight-walk section. |
| Trunk - Backward Sway Max SD | Standard Deviation of the measurements of the sagittal projection of the torso’s maximum backward tilt relative to the gravity vertical across all gait cycles within a straight-walk section. |
| Trunk - Forward Sway Max | Mean of the measurements of the sagittal projection of the torso’s maximum forward tilt relative to the gravity vertical across all gait cycles within a straight-walk section. |
| Trunk - Forward Sway Max SD | Standard Deviation of the sagittal projection of the torso’s maximum forward tilt relative to the gravity vertical across all gait cycles within a straight-walk section. |
| Trunk - Max Transverse Angular Velocity | Mean of the measurements of the transverse projection of the torso’s maximum angular velocity across all gait cycles within a straight-walk section. |
| Trunk - Max Transverse Angular Velocity SD | Standard Deviation of the measurements of the transverse projection of the torso’s maximum angular velocity across all gait cycles within a straight-walk section. |
| Trunk - Right Rotation Max | Mean of the measurements of the transverse projection of the torso’s maximum right turn across all gait cycles within a straight-walk section. |
| Trunk - Right Rotation Max SD | Standard Deviation of the measurements of the transverse projection of the torso’s maximum right turn across all gait cycles within a straight-walk section. |
| Trunk - Left Rotation Max | Mean of the measurements of the transverse projection of the torso’s maximum left turn across all gait cycles within a straight-walk section. |
| Trunk - Left Rotation Max SD | Standard Deviation of the measurements of the transverse projection of the torso’s maximum left turn across all gait cycles within a straight-walk section. |
| Trunk - Sway Max | Mean of the measurements of Trunk - Left Sway Max and Trunk - Right Sway Max in each gait cycle. Finally, the average is taken across all gait cycles within a straight-walk section. |
| Trunk - Sway Max SD | Standard Deviation of all the measurements of Trunk - Left Sway Max and Trunk - Right Sway Max through all the gait cycles within a straight-walk section. |
| Lumbar - Max Coronal Angular Velocity | Mean of the measurements of the coronal projection of the waist’s maximum angular velocity across all gait cycles within a straight-walk section. |
| Lumbar - Max Coronal Angular Velocity SD | Standard Deviation of the measurements of the projection of the waist’s maximum angular velocity across all gait cycles within a straight-walk section. |
| Lumbar - Right Sway Max | Mean of the measurements of the coronal projection of the waist’s maximum right tilt relative to the gravity vertical across all gait cycles within a straight-walk section. |
| Lumbar - Right Sway Max SD | Standard Deviation of the measurements of the coronal projection of the waist’s maximum right tilt relative to the gravity vertical through all the gait cycles within a straight-walk section. |
| Lumbar - Left Sway Max | Mean of the measurements of the coronal projection of the waist’s maximum left tilt relative to the gravity vertical across all gait cycles within a straight-walk section. |
| Lumbar - Left Sway Max SD | Standard Deviation of the measurements of the coronal projection of the waist’s maximum left tilt relative to the gravity vertical through all the gait cycles within a straight-walk section. |
| Lumbar - Max Sagittal Angular Velocity | Mean of the measurements of the sagittal projection of the waist’s maximum angular velocity across all gait cycles within a straight-walk section. |
| Lumbar - Max Sagittal Angular Velocity SD | Standard Deviation of the measurements of the sagittal projection of the waist’s sagittal maximum angular velocity across all gait cycles within a straight-walk section. |
| Lumbar - Forward Sway Max | Mean of the measurements of the sagittal projection of the waist’s maximum forward tilt relative to the gravity vertical across all gait cycles within a straight-walk section. |
| Lumbar - Forward Sway Max SD | Standard Deviation of the measurements of the sagittal projection of the waist’s maximum forward tilt relative to the gravity vertical across all gait cycles within a straight-walk section. |
| Lumbar - Backward Swaying Max | Mean of the measurements of the sagittal projection of the waist’s maximum backward tilt relative to the gravity vertical across all gait cycles within a straight-walk section. |
| Lumbar - Backward Swaying Max SD | Standard Deviation of the sagittal projection of the waist’s maximum backward tilt relative to the gravity vertical across all gait cycles within a straight-walk section. |
| Lumbar - Max Transverse Angular Velocity | Mean of the measurements of the transverse projection of the waist’s maximum angular velocity across all gait cycles within a straight-walk section. |
| Lumbar - Max Transverse Angular Velocity SD | Standard Deviation of the measurements of the transverse projection of the waist’s maximum angular velocity across all gait cycles within a straight-walk section. |
| Lumbar - Right Rotation Max | Mean of the measurements of the transverse projection of the waist’s maximum right turn across all gait cycles within a straight-walk section. |
| Lumbar - Right Rotation Max SD | Standard Deviation of the measurements of the transverse projection of the waist’s maximum right turn across all gait cycles within a straight-walk section. |
| Lumbar - Left Rotation Max | Mean of the measurements of the transverse projection of the waist’s maximum left turn across all gait cycles within a straight-walk section. |
| Lumbar - Left Rotation Max SD | Standard Deviation of the measurements of the transverse projection of the waist’s maximum left turn across all gait cycles within a straight-walk section. |
| Arm - Max Sagittal Angular Velocity R | Mean of the measurements of the right-arm’s maximum angular velocity across all gait cycles within a straight-walk section. |
| Arm - Max Sagittal Angular Velocity R SD | Standard Deviation of the measurements of the right-arm’s maximum angular velocity across all gait cycles within a straight-walk section. |
| Arm - Max Sagittal Angular Velocity L | Mean of the measurements of the left-arm’s maximum angular velocity across all gait cycles within a straight-walk section. |
| Arm - Max Sagittal Angular Velocity L SD | Standard Deviation of the measurements of the lef-arm’s maximum angular velocity across all gait cycles within a straight-walk section. |
| Arm - Max Sagittal Angular Velocity | Mean of Left Arm - Max Sagittal Angular Velocity and Right Arm - Max Sagittal Angular Velocity in each gait cycle. Finally, the average is taken across all gait cycles within a straight-walk section. |
| Arm - Max Sagittal Angular Velocity SD | Standard Deviation of all the measurements of Left Arm - Max Sagittal Angular Velocity and Right Arm - Max Sagittal Angular Velocity through all the gait cycles within a straight-walk section. |
| Arm - Forward Swing Max R | The maximum angle of the forward swing of the right arm within each gait cycle. Finally, the average is taken across all gait cycles within a straight-walk section. |
| Arm - Forward Swing Max R SD | Standard deviation of all maximum forward swing angles of the right arm through all the gait cycles within a straight-walk section. |
| Arm - Forward Swing Max L | The maximum angle of the forward swing of the Left arm within each gait cycle. Finally, the average is taken across all gait cycles within a straight-walk section. |
| Arm - Forward Swing Max L SD | Standard deviation of all maximum forward swing angles of the Left arm through all the gait cycles within a straight-walk section. |
| Arm - Forward Swing Max | Mean of Left Arm - Forward Swing Max and Right Arm - Forward Swing Max in each gait cycle. Finally, the average is taken across all gait cycles within a straight-walk section. |
| Arm - Forward Swing Max SD | Standard Deviation of all the measurements of Left Arm - Forward Swing Max and Right Arm - Forward Swing Max through all the gait cycles within a straight-walk section. |
| Arm - Backward Swing Max R | The maximum angle of the backward swing of the right arm within each gait cycle. Finally, the average is taken across all gait cycles within a straight-walk section. |
| Arm - Backward Swing Max R SD | Standard deviation of all maximum backward swing angles of the right arm through all the gait cycles within a straight-walk section. |
| Arm - Backward Swing Max L | The maximum angle of the backward swing of the Left arm within each gait cycle. Finally, the average is taken across all gait cycles within a straight-walk section. |
| Arm - Backward Swing Max L SD | Standard deviation of all maximum backward swing angles of the Left arm through all the gait cycles within a straight-walk section. |
| Arm - Backward Swing Max | Mean of Left Arm - Backward Swing Max and Right Arm - Backward Swing Max in each gait cycle. Finally, the average is taken across all gait cycles within a straight-walk section. |
| Arm - Backward Swing Max SD | Standard Deviation of all the measurements of Left Arm - Backward Swing Max and Right Arm - Backward Swing Max through all the gait cycles within a straight-walk section. |
| Arm - Asymmetry of Max Sagittal Angular Velocity | Among the pairs of successive Left Gait Cycles and Right Gait Cycles in a straight-walk section, the parameter is calculated as the ratios of the absolute values of the differences between the successive left arm’s Max Sagittal Angular Velocity and the right arm’s Max Sagittal Angular Velocity, to the larger ones of them. Finally, the average is taken across all gait cycles within a straight-walk section. |
| Arm - Asymmetry of Max Sagittal Angular Velocity SD | Standard Deviation of all the measurements of Arm - Asymmetry of Max Sagittal Angular Velocity through all the gait cycles within a straight-walk section. |
| Arm - Difference of Max Sagittal Angular Velocity | Among the pairs of successive Left Gait Cycles and Right Gait Cycles in a straight-walk section, the parameter is calculated as the absolute values of the differences between the successive left arm’s Max Sagittal Angular Velocity and the right arm’s Max Sagittal Angular Velocity. Finally, the average is taken across all gait cycles within a straight-walk section. |
| Arm - Difference of Max Sagittal Angular Velocity SD | Standard Deviation of all the measurements of Arm - Difference of Max Sagittal Angular Velocity through all the gait cycles within a straight-walk section. |
| Arm - Symbolic Symmetry Index | Can be applied to describe the symmetry of arms’ movements in a straight-walk section. In each gait cycle, a measurement of Arm Symbolic Symmetry Index is performed, and finally, the average is taken across all gait cycles within a straight-walk section. An excellent performance means that the parameter approaches zero, while on the contrary, it approaches 100%. |
| Arm - Symbolic Symmetry Index SD | Standard Deviation of all the measurements of Arm - Symbolic Symmetry Index through all the gait cycles within a straight-walk section. |
| **Part2 Segmentation features** |  |
| SW - Trunk - Right Sway Max | The coronal projection of the torso’s maximum right tilt relative to the gravity vertical through straight walk |
| SW - Trunk - Left Sway Max | The coronal projection of the torso’s maximum left tilt relative to the gravity vertical through straight walk |
| SW - Trunk - Coronal Mean Sway | Mean of the measurements of the coronal projection of the torso’s tilt relative to the gravity vertical through straight walk |
| SW - Trunk - Coronal Sway Range | Range of the measurements of the coronal projection of the torso’s tilt relative to the gravity vertical through straight walk |
| SW - Trunk - Coronal Sway Slope | The slope of the smooth curve of the coronal projection of the torso’s tilt relative to the gravity vertical through straight walk |
| SW - Trunk - Difference of Coronal Sway | The difference of the coronal projection of the torso’s tilt relative to the gravity vertical between the start and end moment of the straight walk |
| SW - Trunk - Backward Sway Max | The sagittal projection of the torso’s maximum backward tilt relative to the gravity vertical through straight walk |
| SW - Trunk - Forward Sway Max | The sagittal projection of the torso’s maximum forward tilt relative to the gravity vertical through straight walk |
| SW - Trunk - Sagittal Mean Sway | Mean of the measurements of the sagittal projection of the torso’s tilt relative to the gravity vertical through straight walk |
| SW - Trunk - Sagittal Sway Range | Range of the measurements of the sagittal projection of the torso’s tilt relative to the gravity vertical through straight walk |
| SW - Trunk - Sagittal Sway Slope | The slope of the smooth curve of the sagittal projection of the torso’s tilt relative to the gravity vertical through straight walk |
| SW - Trunk - Difference of Sagittal Sway | The difference of the sagittal projection of the torso’s tilt relative to the gravity vertical between the start and end moment of the straight walk |
| SW - Lumbar - Right Sway Max | The coronal projection of the waist’s maximum right tilt relative to the gravity vertical through straight walk |
| SW - Lumbar - Left Sway Max | The coronal projection of the waist’s maximum left tilt relative to the gravity vertical through straight walk |
| SW - Lumbar - Coronal Mean Sway | Mean of the measurements of the coronal projection of the waist’s tilt relative to the gravity vertical through straight walk |
| SW - Lumbar - Coronal Sway Range | Range of the measurements of the coronal projection of the waist’s tilt relative to the gravity vertical through straight walk |
| SW - Lumbar - Coronal Sway Slope | The slope of the smooth curve of the coronal projection of thewaist’s tilt relative to the gravity vertical through straight walk |
| SW - Lumbar - Difference of Coronal Sway | The difference of the coronal projection of the waist’s tilt relative to the gravity vertical between the start and end moment of the straight walk |
| SW - Lumbar - Backward Sway Max | The sagittal projection of the waist’s maximum backward tilt relative to the gravity vertical through straight walk |
| SW - Lumbar - Forward Sway Max | The sagittal projection of the waist’s maximum forward tilt relative to the gravity vertical through straight walk |
| SW - Lumbar - Sagittal Mean Sway | Mean of the measurements of the sagittal projection of the waist’s tilt relative to the gravity vertical through straight walk |
| SW - Lumbar - Sagittal Sway Range | Range of the measurements of the sagittal projection of the waist’s tilt relative to the gravity vertical through straight walk |
| SW - Lumbar - Sagittal Sway Slope | The slope of the smooth curve of the sagittal projection of the waist’s tilt relative to the gravity vertical through straight walk |
| SW - Lumbar - Difference of Sagittal Sway | The difference of the sagittal projection of the waist’s tilt relative to the gravity vertical between the start and end moment of the straight walk |
| Walk Speed | The average forward walking speed, that is, the ratio of the walking distance over walking duration of a straight-walk section. |
| Straight-Walking Duration | Total time of straight walk |
| 180° Turn - Duration | The turning time for a turning section. |
| 180° Turn - Steps | The steps count for a turning section. |
| 180° Turn - Max Angular Velocity | The maximum absolute value of the torso's rotational angular velocity in the transverse plane during a turning section. |
| 180° Turn - Mean Angular Velocity | The ratio of the torso's rotation angle to the rotation time in the transverse plane during turning is used as the average angular velocity for this turning section. |
| 180° Turn - Step Duration | The time interval between the ground contact of one foot and the ground contact of the opposite foot is defined as the step duration. The average step duration during a turning section is calculated based on these values. |
| 180° Turn - Step Duration SD | Standard Deviation of all the measurements of 180° Turn - Step Duration in a turning section. |
| 180° Turn - Trunk - Right Sway Max | The coronal projection of the torso’s maximum right tilt relative to the gravity vertical through turning process |
| 180° Turn - Trunk - Left Sway Max | The coronal projection of the torso’s maximum left tilt relative to the gravity vertical through turning process |
| 180° Turn - Trunk - Coronal Mean Sway | Mean of the measurements of the coronal projection of the torso’s tilt relative to the gravity vertical through turning process |
| 180° Turn - Trunk - Coronal Sway Range | Range of the measurements of the coronal projection of the torso’s tilt relative to the gravity vertical through turning process |
| 180° Turn - Trunk - Coronal Sway Slope | The slope of the smooth curve of the coronal projection of the torso’s tilt relative to the gravity vertical through turning process |
| 180° Turn - Trunk - Difference of Coronal Sway | The difference of the coronal projection of the torso’s tilt relative to the gravity vertical between the start and end moment of the turning process |
| 180° Turn - Trunk - Backward Sway Max | The sagittal projection of the torso’s maximum backward tilt relative to the gravity vertical through turning process |
| 180° Turn - Trunk - Forward Sway Max | The sagittal projection of the torso’s maximum forward tilt relative to the gravity vertical through turning process |
| 180° Turn - Trunk - Sagittal Mean Sway | Mean of the measurements of the sagittal projection of the torso’s tilt relative to the gravity vertical through turning process |
| 180° Turn - Trunk - Sagittal Sway Range | Range of the measurements of the sagittal projection of the torso’s tilt relative to the gravity vertical through turning process |
| 180° Turn - Trunk - Sagittal Sway Slope | The slope of the smooth curve of the sagittal projection of the torso’s tilt relative to the gravity vertical through turning process |
| 180° Turn - Trunk - Difference of Sagittal Sway | The difference of the sagittal projection of the torso’s tilt relative to the gravity vertical between the start and end moment of the turning process |
| 180° Turn - Lumbar - Right Sway Max | The coronal projection of the waist’s maximum right tilt relative to the gravity vertical through turning process |
| 180° Turn - Lumbar - Left Sway Max | The coronal projection of the waist’s maximum left tilt relative to the gravity vertical through turning process |
| 180° Turn - Lumbar - Coronal Mean Sway | Mean of the measurements of the coronal projection of the waist’s tilt relative to the gravity vertical through turning process |
| 180° Turn - Lumbar - Coronal Sway Range | Range of the measurements of the coronal projection of the waist’s tilt relative to the gravity vertical through turning process |
| 180° Turn - Lumbar - Coronal Sway Slope | The slope of the smooth curve of the coronal projection of thewaist’s tilt relative to the gravity vertical through turning process |
| 180° Turn - Lumbar - Difference of Coronal Sway | The difference of the coronal projection of the waist’s tilt relative to the gravity vertical between the start and end moment of the turning process |
| 180° Turn - Lumbar - Backward Sway Max | The sagittal projection of the waist’s maximum backward tilt relative to the gravity vertical through turning process |
| 180° Turn - Lumbar - Forward Sway Max | The sagittal projection of the waist’s maximum forward tilt relative to the gravity vertical through turning process |
| 180° Turn - Lumbar - Sagittal Mean Sway | Mean of the measurements of the sagittal projection of the waist’s tilt relative to the gravity vertical through turning process |
| 180° Turn - Lumbar - Sagittal Sway Range | Range of the measurements of the sagittal projection of the waist’s tilt relative to the gravity vertical through turning process |
| 180° Turn - Lumbar - Sagittal Sway Slope | The slope of the smooth curve of the sagittal projection of the waist’s tilt relative to the gravity vertical through turning process |
| 180° Turn - Lumbar - Difference of Sagittal Sway | The difference of the sagittal projection of the waist’s tilt relative to the gravity vertical between the start and end moment of the turning process |
| 180° Turn -Total Duration | Total time of turing process |
| **Part3 Whole trail features** |  |
| WT - Trunk - Coronal Start Sway | The coronal projection of the torso’s tilt relative to the gravity vertical at the start moment of the whole trail |
| WT - Trunk - Coronal End Sway | The coronal projection of the torso’s tilt relative to the gravity vertical at the end moment of the whole trail |
| WT - Trunk - Sagittal Start Sway | The sagittal projection of the torso’s tilt relative to the gravity vertical at the start moment of the whole trail |
| WT - Trunk - Sagittal End Sway | The sagittal projection of the torso’s tilt relative to the gravity vertical at the end moment of the whole trail |
| WT - Lumbar - Coronal Start Sway | The coronal projection of the waist’s tilt relative to the gravity vertical at the start moment of the whole trail |
| WT - Lumbar - Coronal End Sway | The coronal projection of the waist’s tilt relative to the gravity vertical at the end moment of the whole trail |
| WT - Lumbar - Sagittal Start Sway | The sagittal projection of the waist’s tilt relative to the gravity vertical at the start moment of the whole trail |
| WT - Lumbar - Sagittal End Sway | The sagittal projection of the waist’s tilt relative to the gravity vertical at the end moment of the whole trail |
| WT - Trunk - Difference of Coronal Sway | Difference between 'WT - Trunk - Coronal Start Sway' and 'WT - Trunk - Coronal End Sway' |
| WT - Trunk - Difference of Sagitta Sway | Difference between WT - Trunk - Sagittal Start Sway' and 'WT - Trunk - Sagittal End Sway' |
| WT - Lumbar - Difference of Coronal Sway | Difference between 'WT - Lumbar - Coronal Start Sway' and 'WT - Lumbar - Coronal End Sway' |
| WT - Lumbar - Difference of Sagitta Sway | Difference between 'WT - Lumbar - Sagittal Start Sway' and 'WT - Lumbar - Sagittal End Sway' |
| Effective Duration | Effective duration of the gait assessment |

Note. Abbreviations: GCT: Gait Cycle Time; SD: Standard deviation

**eTable 2 Features for model construction on item # 3.9 (arising from chair)**

| **Feature** | **p*** | **R^a^** | **Gain^b^** |
| --- | --- | --- | --- |
| Shank - Swing RoM - max (max) (degree) | 1.426E-11 | -0.476 | 0.121 |
| Stride Variability - min (%) | 9.34E-10 | 0.436 | 0.054 |
| 180° Turn - Mean Angular Velocity - max (degree/s) | 1.086E-12 | -0.498 | 0.035 |
| Stride Velocity Asymmetry SD - max (%) | 0.1714 | 0.102 | 0.028 |
| 180° Turn - Lumbar - Difference of Coronal Sway - max (degree) | 0.02398 | -0.168 | 0.026 |
| 180° Turn - Trunk - Sway Max - max (degree) | 0.6039 | 0.039 | 0.025 |
| Stride Velocity Difference - max (m/s) | 2.248E-09 | -0.427 | 0.02 |
| Effective Duration - (s) | 2.394E-09 | 0.426 | 0.019 |
| 180° Turn - Max Angular Velocity - max (degree/s) | 1.731E-12 | -0.494 | 0.018 |
| Arm - Max Sagittal Angular Velocity SD - max (diff) (degree/s) | 0.4999 | 0.051 | 0.017 |
| SW - Lumbar - Sagittal Sway Range - max (degree) | 0.1537 | -0.107 | 0.017 |
| Stride Length Difference - max (cm) | 1.398E-09 | -0.432 | 0.016 |
| Trunk - Max Sagittal Angular Velocity - min (degree/s) | 4.034E-05 | -0.301 | 0.015 |
| SW - Lumbar - Backward Sway Max - max (degree) | 0.04565 | -0.149 | 0.014 |
| SW - Lumbar - Sagittal Sway Range - mean (degree) | 0.1176 | -0.117 | 0.013 |
| Shank - Forward Swing Max SD - max (max) (degree) | 0.6711 | -0.032 | 0.013 |
| Arm - Difference of Max Sagittal Angular Velocity - max (degree/s) | 0.007293 | -0.199 | 0.011 |
| Stance - max (max) (%GCT) | 9.342E-06 | 0.324 | 0.011 |
| WT - Trunk - Difference of Coronal Sway - mean (degree) | 0.003989 | -0.214 | 0.01 |
| Trunk - Rotation Max - min (max) (degree) | 0.002208 | -0.227 | 0.01 |
| Shank - Swing RoM - min (diff) (degree) | 0.3923 | -0.064 | 0.01 |
| 180° Turn - Trunk - Sagittal Sway Range - max (degree) | 0.04668 | -0.148 | 0.01 |
| Effective Trial Duration - (s) | 2.626E-11 | 0.471 | 0.009 |
| 180° Turn - Trunk - Coronal Mean Sway - min (degree) | 0.3747 | 0.067 | 0.009 |
| 180° Turn - Trunk - Coronal Sway Range - min (degree) | 0.9993 | 0 | 0.008 |
| 180° Turn - Lumbar - Difference of Sagittal Sway - min (degree) | 0.0001915 | 0.275 | 0.008 |
| WT - Lumbar - Difference of Sagitta Sway - mean (degree) | 0.04477 | 0.15 | 0.008 |
| Arm - Symbolic Symmetry Index - mean (%) | 0.704 | 0.029 | 0.008 |
| Swing SD - max (max) (%GCT) | 0.001127 | 0.241 | 0.008 |
| 180° Turn - Step Duration - min (s) | 0.1052 | 0.121 | 0.007 |
| Arm - Swing RoM - max (max) (degree) | 0.0007326 | -0.249 | 0.007 |
| Lumbar - Rotation Max - max (max) (degree) | 4.824E-05 | -0.298 | 0.007 |
| WT - Lumbar - Sagittal Start Sway - mean (degree) | 0.2754 | -0.082 | 0.007 |
| Lumbar - Backward Swaying Max - mean (degree) | 0.1395 | -0.111 | 0.007 |
| SW - Trunk - Sway Max - mean (degree) | 0.2094 | 0.094 | 0.007 |

Note. GCT: Gait cycle time, ROM: Range of motion;

^a^R was the spearman correlation coefficient between the corresponding feature and scores on item #3.9

^b^Gain was the relative contribution of the corresponding feature to the predictive model

^*^p was estimated whether the spearman correlation between the corresponding feature and scores on item #3.9 significant or not

**eTable 3 Features for model construction on item # 3.10 (gait)**

| **Feature** | **p*** | **R^a^** | **Gain^b^** |
| --- | --- | --- | --- |
| Shank - Swing RoM - mean (max) (degree) | 3.203E-21 | -0.629 | 0.163 |
| Shank - Max Sagittal Angular Velocity - max (degree/s) | 1.823E-17 | -0.578 | 0.064 |
| Shank - Swing RoM - min (max) (degree) | 3.302E-20 | -0.616 | 0.041 |
| Trunk - Max Sagittal Angular Velocity - mean (degree/s) | 5.876E-08 | -0.391 | 0.035 |
| 180° Turn - Lumbar - Coronal Sway Range - min (degree) | 0.8367 | -0.015 | 0.028 |
| Swing SD - max (%GCT) | 0.00008252 | 0.289 | 0.026 |
| Lumbar - Rotation Max - mean (min) (degree) | 0.1313 | 0.113 | 0.025 |
| Lumbar - Rotation Max - min (diff) (degree) | 0.144 | -0.109 | 0.025 |
| Shank - Swing RoM - max (diff) (degree) | 0.2002 | -0.096 | 0.021 |
| Arm - Swing RoM - max (max) (degree) | 2.946E-08 | -0.399 | 0.021 |
| Swing SD - max (min) (%GCT) | 1.61E-10 | 0.454 | 0.017 |
| Shank - Max Sagittal Angular Velocity - mean (degree/s) | 3.179E-17 | -0.575 | 0.017 |
| Shank - Swing Speed SD - max (diff) (m/s) | 0.01202 | -0.187 | 0.016 |
| Arm - Symbolic Symmetry Index SD - max (%) | 0.06127 | -0.14 | 0.015 |
| Phase Coordination Index - mean (%) | 0.00001381 | 0.318 | 0.013 |
| Coordination - min (%) | 0.004905 | 0.209 | 0.012 |
| Stride Variability - max (%) | 0.00007242 | 0.291 | 0.012 |
| 180° Turn - Steps SD - max (#) | 0.0188 | 0.175 | 0.011 |
| 180° Turn - Lumbar - Sagittal Sway Slope - mean | 4.783E-12 | 0.486 | 0.011 |
| Lumbar - Max Coronal Angular Velocity SD - max (degree/s) | 3.075E-08 | -0.398 | 0.01 |
| Lumbar - Max Transverse Angular Velocity - mean (degree/s) | 0.001381 | -0.237 | 0.01 |
| Shank - Forward Swing Max - mean (degree) | 4.325E-17 | -0.573 | 0.009 |
| Lumbar - Sway Max SD - max (min) (degree) | 0.5131 | 0.049 | 0.009 |
| SW - Lumbar - Sagittal Sway Slope - min | 1.884E-08 | -0.404 | 0.009 |
| Shank - RoM Absolute Difference - max (degree) | 0.3209 | -0.074 | 0.008 |
| Trunk - Sway Max SD - max (diff) (degree) | 0.0002175 | -0.272 | 0.008 |
| 180° Turn - Steps - mean (#) | 1.549E-13 | 0.514 | 0.008 |
| Stance - max (max) (%GCT) | 0.00003114 | 0.305 | 0.008 |
| Gait Cycle - mean (diff) (s) | 0.9289 | -0.007 | 0.008 |
| 180° Turn - Lumbar - Sagittal Sway Range - mean (degree) | 2.763E-08 | -0.4 | 0.007 |
| Arm - Max Sagittal Angular Velocity - min (degree/s) | 1.503E-08 | -0.406 | 0.007 |
| Effective Trial Duration - (s) | 6.043E-14 | 0.522 | 0.007 |
| Lumbar - Max Coronal Angular Velocity - mean (degree/s) | 2.942E-08 | -0.399 | 0.007 |
| Cadence SD - max (max) (step/min) | 0.00000198 | 0.346 | 0.007 |
| SW - Lumbar - Coronal Sway Range - min (degree) | 0.03946 | -0.154 | 0.007 |

Note. GCT: Gait cycle time, ROM: Range of motion;

^a^R was the spearman correlation coefficient between the corresponding feature and scores on item #3.10

^b^Gain was the relative contribution of the corresponding feature to the predictive model

^*^p was estimated whether the spearman correlation between the corresponding feature and scores on item #3.10 significant or not

**eTable 4 Features for model construction on item #3.11 (freezing of gait)**

| **Feature** | **p*** | **R^a^** | **Gain^b^** |
| --- | --- | --- | --- |
| 180° Turn - Steps - mean (#) | 7.623E-12 | 0.482 | 0.164 |
| 180° Turn - Lumbar - Coronal Sway Slope - max | 0.0000258 | -0.308 | 0.067 |
| Gait Cycle SD - max (max) (s) | 0.0002558 | 0.269 | 0.037 |
| Shank - Backward Swing Max - mean (degree) | 4.658E-07 | 0.365 | 0.035 |
| Arm - Max Sagittal Angular Velocity - min (min) (degree/s) | 0.0001154 | -0.283 | 0.033 |
| Shank - Max Sagittal Angular Velocity SD - max (degree/s) | 0.7948 | 0.02 | 0.033 |
| Double Support - mean (diff) (%GCT) | 0.7568 | 0.023 | 0.029 |
| Trunk - Max Coronal Angular Velocity - min (degree/s) | 0.02349 | -0.169 | 0.018 |
| 180° Turn - Trunk - Coronal Sway Range - min (degree) | 0.2486 | -0.086 | 0.018 |
| Straight-Walking Duration - min (s) | 8.282E-07 | 0.358 | 0.017 |
| SW - Trunk - Difference of Sagittal Sway - mean (degree) | 0.01235 | -0.186 | 0.016 |
| Cadence SD - max (max) (step/min) | 9.114E-05 | 0.287 | 0.016 |
| Shank - Forward Swing Max SD - max (max) (degree) | 0.141 | 0.11 | 0.016 |
| Shank - Max Sagittal Angular Velocity - mean (min) (degree/s) | 0.000517 | -0.256 | 0.014 |
| 180° Turn - Duration - min (s) | 3.463E-06 | 0.338 | 0.013 |
| Stance - max (diff) (%GCT) | 0.4432 | 0.058 | 0.012 |
| Trunk - Sway Max - max (degree) | 0.0002274 | -0.271 | 0.012 |
| Arm - Backward Swing Max SD - max (degree) | 0.007254 | 0.199 | 0.012 |
| Shank - Swing Speed - min (diff) (m/s) | 0.635 | -0.036 | 0.012 |
| 180° Turn - Steps SD - max (#) | 3.251E-06 | 0.339 | 0.011 |
| SW - Trunk - Forward Sway Max - mean (degree) | 0.000586 | -0.254 | 0.011 |
| 180° Turn - Trunk - Sagittal Sway Slope - min | 0.09126 | 0.126 | 0.011 |
| Shank - RoM Asymmetry SD - max (%) | 0.001355 | 0.237 | 0.01 |
| Stride Length - mean (diff) (cm) | 0.1263 | -0.114 | 0.01 |
| Arm - Backward Swing Max SD - max (max) (degree) | 0.04815 | 0.148 | 0.01 |
| Trunk - Backward Sway Max - mean (degree) | 2.285E-05 | -0.31 | 0.01 |
| WT - Lumbar - Difference of Coronal Sway - mean (degree) | 0.4155 | 0.061 | 0.009 |
| Arm - Max Sagittal Angular Velocity SD - max (min) (degree/s) | 0.1362 | -0.111 | 0.008 |
| Shank - Forward Swing Max SD - max (diff) (degree) | 0.5072 | 0.05 | 0.008 |
| Trunk - Max Transverse Angular Velocity SD - max (degree/s) | 0.01049 | -0.19 | 0.008 |

Note. GCT: Gait cycle time, ROM: Range of motion;

^a^R was the spearman correlation coefficient between the corresponding feature and scores on item #3.11

^b^Gain was the relative contribution of the corresponding feature to the predictive model

^*^p was estimated whether the spearman correlation between the corresponding feature and scores on item #3.11 significant or not

**eTable 5 Features for model construction on item #** **3.12 (postural stability)**

| **Feature** | **p*** | **R^a^** | **Gain^b^** |
| --- | --- | --- | --- |
| Shank - Swing RoM - max (max) (degree) | 1.704E-17 | -0.579 | 0.059 |
| 180° Turn - Duration - mean (s) | 3.049E-19 | 0.604 | 0.035 |
| Arm - Max Sagittal Angular Velocity SD - max (min) (degree/s) | 0.04836 | -0.147 | 0.034 |
| Double Support SD - max (max) (%GCT) | 0.004975 | 0.208 | 0.022 |
| SW - Lumbar - Coronal Sway Slope - min | 1.091E-12 | 0.498 | 0.022 |
| 180° Turn - Max Angular Velocity - max (degree/s) | 5.716E-18 | -0.586 | 0.02 |
| Gait Speed - max (diff) (m/s) | 0.00304 | -0.22 | 0.018 |
| Shank - Swing RoM - mean (diff) (degree) | 0.1476 | -0.108 | 0.018 |
| Lumbar - Max Sagittal Angular Velocity - max (degree/s) | 0.000465 | -0.258 | 0.017 |
| Shank - Swing RoM - min (diff) (degree) | 0.6151 | 0.038 | 0.017 |
| Swing - mean (diff) (%GCT) | 0.04018 | 0.153 | 0.015 |
| Arm - Max Sagittal Angular Velocity - max (max) (degree/s) | 0.00001256 | -0.319 | 0.013 |
| Lumbar - Rotation Max SD - max (min) (degree) | 0.01694 | 0.178 | 0.013 |
| Arm - Forward Swing Max SD - max (diff) (degree) | 0.3686 | -0.067 | 0.013 |
| Stride Velocity Difference SD - max (m/s) | 0.2922 | 0.079 | 0.013 |
| Trunk - Rotation Max - max (min) (degree) | 0.5713 | 0.042 | 0.012 |
| Gait Speed - max (max) (m/s) | 1.721E-16 | -0.564 | 0.012 |
| Arm - Forward Swing Max SD - max (degree) | 0.7313 | -0.026 | 0.012 |
| Trunk - Sway Max SD - max (max) (degree) | 0.829 | -0.016 | 0.011 |
| Shank - Max Sagittal Angular Velocity - max (min) (degree/s) | 8.879E-13 | -0.5 | 0.011 |
| Shank - Backward Swing Max - min (degree) | 6.084E-10 | 0.441 | 0.011 |
| Straight-Walking Duration - mean (s) | 1.055E-15 | 0.551 | 0.011 |
| Step Length - max (min) (cm) | 1.907E-14 | -0.53 | 0.01 |
| Swing - max (%GCT) | 0.00001292 | -0.319 | 0.01 |
| SW - Trunk - Sway Max - max (degree) | 0.04627 | -0.149 | 0.01 |
| Trunk - Backward Sway Max - max (degree) | 0.00001085 | -0.321 | 0.01 |
| Lumbar - Forward Sway Max SD - max (degree) | 0.2045 | -0.095 | 0.009 |
| Arm - Asymmetry of Max Sagittal Angular Velocity SD - max (%) | 0.3467 | -0.071 | 0.009 |
| 180° Turn - Lumbar - Coronal Sway Range - max (degree) | 0.003405 | -0.217 | 0.009 |
| Arm - Max Sagittal Angular Velocity SD - max (max) (degree/s) | 0.1074 | -0.12 | 0.009 |

Note. GCT: Gait cycle time, ROM: Range of motion;

^a^R was the spearman correlation coefficient between the corresponding feature and scores on item #3.12

^b^Gain was the relative contribution of the corresponding feature to the predictive model

^*^p was estimated whether the spearman correlation between the corresponding feature and scores on item #3.12 significant or not

**eTable 6 Features for model construction on item #** **3.13 (posture)**

| **Feature** | **p*** | **R^a^** | **Gain^b^** |
| --- | --- | --- | --- |
| SW - Lumbar - Difference of Sagittal Sway - max (degree) | 8.107E-11 | -0.46 | 0.054 |
| 180° Turn - Trunk - Backward Sway Max - min (degree) | 1.354E-14 | -0.533 | 0.049 |
| 180° Turn - Trunk - Backward Sway Max - max (degree) | 1.511E-14 | -0.532 | 0.037 |
| Lumbar - Max Transverse Angular Velocity SD - max (degree/s) | 0.1211 | -0.116 | 0.035 |
| Double Support SD - max (diff) (%GCT) | 0.974 | -0.002 | 0.024 |
| 180° Turn -Total Duration - mean (s) | 5.088E-15 | 0.54 | 0.023 |
| Trunk - Backward Sway Max - mean (degree) | 3.231E-12 | -0.489 | 0.019 |
| 180° Turn - Steps SD - max (#) | 0.01596 | 0.179 | 0.017 |
| 180° Turn - Steps - mean (#) | 4.809E-12 | 0.486 | 0.017 |
| Cadence SD - max (max) (step/min) | 0.3366 | 0.072 | 0.017 |
| Trunk - Forward Sway Max - max (degree) | 7.625E-11 | -0.461 | 0.016 |
| Lumbar - Rotation Max - max (diff) (degree) | 0.0007781 | -0.248 | 0.015 |
| Effective Trial Duration - (s) | 3.144E-12 | 0.489 | 0.015 |
| Trunk - Max Sagittal Angular Velocity - max (degree/s) | 3.145E-12 | -0.489 | 0.015 |
| Shank - Swing Speed SD - max (diff) (m/s) | 0.05326 | -0.144 | 0.014 |
| WT - Trunk - Sagittal Start Sway - mean (degree) | 1.034E-11 | -0.479 | 0.014 |
| 180° Turn - Trunk - Sagittal Mean Sway - mean (degree) | 6.81E-14 | -0.521 | 0.013 |
| Stride Length Asymmetry SD - max (%) | 0.009062 | 0.194 | 0.013 |
| Arm - Asymmetry of Max Sagittal Angular Velocity - max (%) | 0.7959 | -0.019 | 0.012 |
| Stance - max (max) (%GCT) | 0.8149 | 0.018 | 0.012 |
| Trunk - Max Sagittal Angular Velocity - min (degree/s) | 2.425E-12 | -0.492 | 0.012 |
| Arm - Forward Swing Max - max (degree) | 0.00121 | -0.239 | 0.012 |
| SW - Trunk - Backward Sway Max - mean (degree) | 3.035E-14 | -0.527 | 0.011 |
| Arm - Asymmetry of Max Sagittal Angular Velocity SD - max (%) | 0.01622 | 0.179 | 0.011 |
| Arm - Forward Swing Max SD - max (min) (degree) | 0.5283 | -0.047 | 0.011 |
| Shank - Symbolic Symmetry Index SD - max (%) | 0.3431 | -0.071 | 0.01 |
| SW - Lumbar - Difference of Coronal Sway - max (degree) | 0.1903 | -0.098 | 0.01 |
| Double Support SD - max (%GCT) | 0.01202 | 0.187 | 0.01 |
| Cadence SD - max (min) (step/min) | 0.0001689 | 0.277 | 0.009 |
| Swing SD - max (max) (%GCT) | 0.0001957 | 0.274 | 0.009 |
| Arm - Forward Swing Max SD - max (diff) (degree) | 0.6229 | 0.037 | 0.009 |
| Stride Length Difference - max (cm) | 3.702E-05 | -0.302 | 0.009 |
| Trunk - Max Sagittal Angular Velocity - mean (degree/s) | 1.755E-12 | -0.494 | 0.009 |
| Lumbar - Max Coronal Angular Velocity SD - max (degree/s) | 3.065E-07 | -0.37 | 0.008 |
| SW - Trunk - Sway Max - mean (degree) | 0.05266 | -0.145 | 0.008 |
| Shank - Asymmetry of Max Sagittal Angular Velocity - max (%) | 0.8545 | 0.014 | 0.008 |
| Coordination - min (%) | 0.2332 | 0.089 | 0.007 |
| 180° Turn - Lumbar - Difference of Coronal Sway - min (degree) | 0.9339 | -0.006 | 0.007 |
| Step Length SD - max (diff) (cm) | 0.003663 | -0.216 | 0.007 |
| 180° Turn - Mean Angular Velocity SD - max (degree/s) | 1.538E-05 | -0.316 | 0.007 |

Note. GCT: Gait cycle time, ROM: Range of motion;

^a^R was the spearman correlation coefficient between the corresponding feature and scores on item #3.13

^b^Gain was the relative contribution of the corresponding feature to the predictive model

^*^p was estimated whether the spearman correlation between the corresponding feature and scores on item #3.13 significant or not

**eTable 7 Contribution of different sensors to each gait and posture item model**

| **Item, # (description)** | **fea_num^a^** | **foot** | **hand** | **thigh** | **shank** | **Lumbar** | **chest** |
| --- | --- | --- | --- | --- | --- | --- | --- |
| 3.9 (arising from chair) | 35 | 8.6% | 17.1% | 17.1% | 31.4% | 28.6% | 37.1% |
| 3.10 (gait) | 35 | 8.6% | 11.4% | 8.6% | 48.6% | 34.3% | 8.6% |
| 3.11 (freezing of gait) | 30 | 6.7% | 13.3% | 6.7% | 43.3% | 10.0% | 30.0% |
| 3.12 (postural stability) | 30 | 0.0% | 20.0% | 13.3% | 43.3% | 20.0% | 20.0% |
| 3.13 (posture) | 40 | 7.5% | 15.0% | 10.0% | 35.0% | 20.0% | 32.5% |

Note. ^a^fea_num: the number of features included in the models. As some features were calculated based on more than one sensor, the cumulative sensor contributions for some gait and posture items were over 100%.

**Reference**

1. Cai G, Shi W, Wang Y, et al. (2023). Specific Distribution of Digital Gait Biomarkers in Parkinson’s Disease Using Body-Worn Sensors and Machine Learning. The Journals of Gerontology: Series A. 78, 1348–1354. DOI: doi.org/10.1093/gerona/glad101
